# Iron Assimilation during Emerging Infections Caused by Opportunistic Fungi with emphasis on Mucorales and the Development of Antifungal Resistance

**DOI:** 10.3390/genes11111296

**Published:** 2020-10-30

**Authors:** Felicia Adelina Stanford, Kerstin Voigt

**Affiliations:** 1 Jena Microbial Resource Collection, Leibniz Institute for Natural Product Research, and Infection Biology–Hans Knöll Institute, Jena, Adolf-Reichwein-Straße 23, 07745 Jena, Germany; felicia.stanford@leibniz-hki.de; 2 Institute of Microbiology, Faculty of Biological Sciences, Friedrich-Schiller University Jena, Neugasse 25, 07743 Jena, Germany; 3 Leibniz Institute for Natural Product Research and Infection Biology–Hans Knöll Institute, Jena Microbial Resource Collection Adolf-Reichwein-Straße 23, 07745 Jena, Germany

**Keywords:** fungal pathogens, fungal infection, metal homeostasis, antifungal resistance, zygomycetes, mucoromycotina, mucoromycetes, *Mucor*, *Rhizopus*, *Lichtheimia*

## Abstract

Iron is a key transition metal required by most microorganisms and is prominently utilised in the transfer of electrons during metabolic reactions. The acquisition of iron is essential and becomes a crucial pathogenic event for opportunistic fungi. Iron is not readily available in the natural environment as it exists in its insoluble ferric form, i.e., in oxides and hydroxides. During infection, the host iron is bound to proteins such as transferrin, ferritin, and haemoglobin. As such, access to iron is one of the major hurdles that fungal pathogens must overcome in an immunocompromised host. Thus, these opportunistic fungi utilise three major iron acquisition systems to overcome this limiting factor for growth and proliferation. To date, numerous iron acquisition pathways have been fully characterised, with key components of these systems having major roles in virulence. Most recently, proteins involved in these pathways have been linked to the development of antifungal resistance. Here, we provide a detailed review of our current knowledge of iron acquisition in opportunistic fungi, and the role iron may have on the development of resistance to antifungals with emphasis on species of the fungal basal lineage order Mucorales, the causative agents of mucormycosis.

## 1. Introduction

In biology, iron is an essential micronutrient for almost all eukaryotes and most prokaryotes [1]. Iron is the fourth most abundant trace element in the environment, but the bioavailability (Fe^2+^) is limited due to oxidation into the insoluble ferric hydroxides (Fe^3+^) by atmospheric oxygen [2]. In this state, iron has a solubility of approximately 10^−9^ M at neutral pH [3]. Nonetheless, the involvement of iron in numerous important metabolic processes and as enzyme cofactors is due to its capacity for electron exchange [4]. This transition metal is required in DNA, RNA and amino acid synthesis, oxygen transport, cellular respiration (iron-sulphur cluster (Fe-S) containing ferredoxins, haem-containing cytochromes), enzymatic reactions such as Fe-S proteins, e.g., fumarase and aconitase of the tricarboxylic acid cycle (TCA cycle) [5,6,7]. Although it is a key trace element, iron also presents a danger to biological systems. Iron (Fe^2+^) triggered Fenton reaction produces reactive oxygen species (ROS) such as superoxide (O_2_•^−^), hydrogen peroxide (H_2_O_2_), and hydroxyl radicals (OH•) (Equation (1)) [8]. Hydroxyl radicals produced during these reactions are deleterious and can damage cellular components such as DNA, proteins, and lipids [9]. Due to the redox property of iron, it is imperative that organisms have tightly regulated homeostatic mechanisms to maintain enough intracellular iron while actively avoiding the detrimental effects of excess iron [10].

Fe^3^ + O_2_• → Fe^2^ + O_2_ Fe^2+^ + H_2_O_2_ → Fe^3+^ + OH• + OH^−^ Net Reaction: O_2_•^−^ + H_2_O_2_ → OH• + OH^−^ + O_2_ (1)

In low iron environments, cells employ strict iron usage called the iron-sparing response, which allows small concentrations to be used in essential enzymatic processes [11]. High-affinity acquisition systems are expressed under these conditions, which allows for the rapid and efficient uptake of iron [3,12]. Under high-iron conditions, these uptake systems are repressed, and excess iron is stored in intracellular compartments, e.g., vacuole or ferritin in mucoralean fungi [3,13,14,15].

In the host, iron is kept extremely low (i.e., <10^−24^ M for Fe^3+^ in serum), and other trace metals, are usually bound to proteins [16]. During infection, iron is further restricted by numerous host mechanisms [17]. These mechanisms function by actively chelating extracellular Fe^3+^ to high-affinity iron-binding proteins such as glycoproteins, transferrin, and lactoferrin, including intracellular sequestration by haemoglobin, ferritin, cytochromes, and the hepcidin axis, to name a few [2,18]. These elegant pathways and mechanisms for controlling systemic iron concentrations are known as nutritional immunity, and its importance in the host immune response to infections has been thoroughly described [2,17,19]. 

Invading fungal pathogens must overcome these limitations to access host iron and other key metals such as zinc, copper, manganese, and nickel to proliferate and cause disease. As such, healthy individuals are usually not susceptible as their immune system is robust [17]. On the other hand, fungal pathogens can cause debilitating and devasting diseases to various patient groups, especially among those who are immunocompromised or hospitalised with severe underlying conditions [20,21]. Those at high risk include patients undergoing haematopoietic stem cell (HSCT), solid organ transplant recipients (SOTs), AIDS patients, those receiving antilymphocyte monoclonal antibodies, and other immunomodulators, as well as patients with other underlying diseases associated with immune dysfunction [20,21]. Opportunistic fungal infections are underappreciated in comparison to bacterial, viral, and parasitic infections [22]. With the current advancements in medicine and the increasing cohort of immunosuppressed individuals, the mortality rate caused by fungal infections is on a constant rise [23]. For instance, *Candida albicans* and other *Candida* species are the most common fungal pathogens responsible for superficial mucosal infections as well as life-threatening systemic diseases [24]. *Cryptococcus neoformans* is the most important opportunistic pathogen in HIV/AIDS patients. Although access to antiretroviral therapy (ART) has improved globally, the number of cryptococcal infections remains high, with an estimated 278,000 reported cases worldwide and a mortality rate of approximately 81% [24,25,26,27,28]. *Aspergillus fumigatus* and other pathogenic *Aspergillus* species cause a wide spectrum of diseases known as aspergilloses. These include allergic bronchopulmonary, chronic pulmonary, and invasive aspergillosis [29]. As fungi cause serious opportunistic infections, there is a new precedent for novel approaches in treatment options, as the range remains limited and there are increasing reports of resistance [30]. 

In this review, we aim to highlight the most recent advancements in our understanding of iron acquisition and metabolism in fungi: (1) the reductive pathway, (2) haem and haemoglobin utilisation (including transferrin, ferritin, and lactoferrin), and (3) ferric iron acquisition from siderophores. Our expanding knowledge in Mucorales will be briefly updated. In addition, we will also explore the role of iron in antifungal therapies as well as innate and emerging resistance to current first-line therapies. Recently, this area has received renewed interest, as iron assimilation is linked to the response to antifungal treatment in the Mucorales [31,32,33]. 

## 2. The Reductive System for Iron Uptake

**Figure 1:**
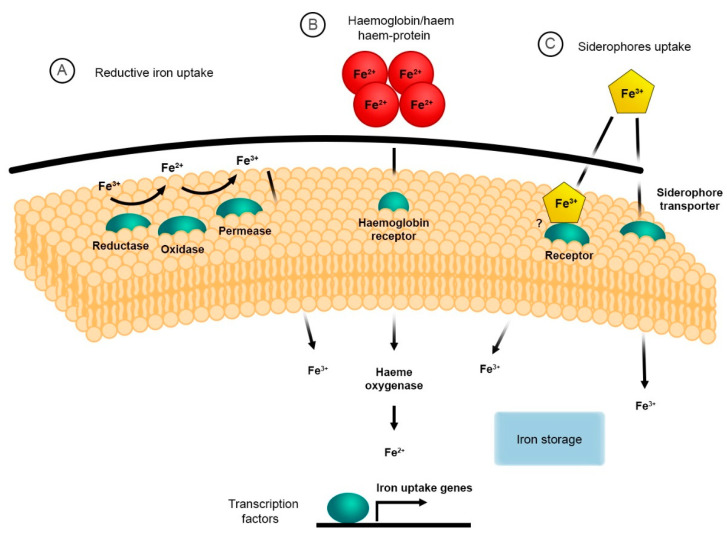
General strategies for iron acquisition in pathogenic fungi. (**A**) the reductive system responsible for iron assimilation via reduction and oxidation followed by transport into the cytoplasm via specialised iron permeases; (**B**) haem -iron uptake and degradation, which facilitates iron chelation from haemoglobin and haem -proteins; (**C**) siderophore uptake system that allows for iron acquisition from a spectrum of siderophores and xenosiderophores (figure adapted from [40]).

The mechanism for iron acquisition and homeostasis has been well documented in the model organism *Saccharomyces cerevisiae*, which established the foundations for further studies in fungal pathogens [34]. There are two main mechanisms for iron uptake in *S. cerevisiae*, the reductive high affinity (HA) and non-reductive systems [35,36]. The reductive HA pathway involves three sequential steps: (i) the initial reduction of ferric (Fe^3+^) to ferrous (Fe^2+^) iron by a dedicated membrane-bound ferric reductase encoded by *FRE1* and *FRE2* genes; (ii) the re-oxidation to ferric iron (Fe^3+^) by the multicopper ferroxidase (ferroxidase) encoded by the *FET3* gene; and (iii) the import of the insoluble ferric iron (Fe^3+^) by the high-affinity iron permease encoded by the *FTR1* gene [37,38,39] (Figure 1). The non-reductive system involves the use of siderophores (xenosiderophores) that bind iron, which are then translocated across the membrane via specific/specialised transporters. This will be discussed later [37,40,41]. 

Fungal pathogens such as *C. neoformans*, *C. albicans* and *A. fumigatus* as well as pathogenic Mucorales, i.e., *Rhizopus arrhizus* (syn. *R. oryzae*, *R. delemar*), *Mucor circinelloides* and *Lichtheimia corymbifera* possess a reductive iron uptake system [42]. This system has highly conserved orthologs of the three major components, i.e., surface ferric reductases, ferroxidases and permeases similar to those identified in *S. cerevisiae* [34,35,43]. For these pathogens, the reductive HA pathway is important for releasing ferric iron bound to other complexes, e.g., transferrin, ferritin, or siderophores [44,45,46,47,48]. The latter organism, *L. corymbifera*, has recently been shown to have conserved orthologs belonging to this system [49]. It has been demonstrated that the ferric reductases are also involved in intracellular iron transport and storage of iron when present on the vacuole membranes [50,51]. The ferric reductases encoded by the *FRE* genes are integral membrane proteins that require NADPH, flavin mononucleotides (FMN), and haem for their activity. The oxidation of cytoplasmic NADPH is catalysed by these ferric reductases, which then transfer the electron across the plasma membrane to facilitate the reduction of metals, e.g., iron [52,53,54]. It has also been shown that these reductases have cupric reductase activity, and they can facilitate the use of siderophore-bound iron [35,44,45,52,55]. Eight putative ferric reductases have been identified in *C. neoformans*; these are *FRE1*–*FRE*7 and *FRE201* [52]. The transcription of *FRE2* and *FRE4* is regulated by FeCl_3_ or haemin, which indicates that these genes may have an important role in iron homeostasis during iron-starvation. Saika et al., 2014 demonstrated that Fre2 is essential for fungal growth in the presence of transferrin and haem and contributed to virulence in mouse inhalation model of cryptococcosis [52]. Copper also plays a role in the transcriptional regulation of the *FRE* genes in *C. neoformans*, *C. albicans*, and *S. cerevisiae* [52,53,56,57,58]. 

As previously mentioned, the next stage in the reductive iron uptake system involves the transport of the reduced iron by the high-affinity ferric transporters. This transport system requires the dual-protein complex consisting of the ferroxidase Fet3 and the permease Ftr1. The ferroxidase, Fet3, catalyses the oxidation of ferrous (Fe^2+^) to ferric iron (Fe^3+^), which is immediately transported into the cell by the permease Ftr1 [59]. Components of the reductive iron uptake system have been identified and characterised in numerous opportunistic fungal pathogens, most of which are thoroughly summarised in the following review [60]. Characterised and putative homologs of the reductive pathway components have been identified in pathogenic Mucorales and are summarised in Table 1. To date, five genes—*FET3*, *FET31*, *FET33*, *FET34,* and *FET99*—have been identified in *C. albicans* that are orthologs to the *S. cerevisiae FET3* gene [44,61]. Under iron starvation, it has been shown that both *FET3* and *FET34* and the permease *FTR1* are regulated. *FET34* has an important role in iron acquisition, hyphal growth, and virulence in murine models of systemic candidiasis [62]. Ftr1 and the ferric reductase Fre10 may be involved in iron acquisition from host proteins, i.e., ferritin and transferrin [45,63,64]. Interestingly, virulence in a mouse model of systemic candidiasis is attenuated in *FTR1* knockouts, and this strongly indicates that the permeases are key virulence determinants [3,44,45,63]. 

The components of the reductive iron uptake system are also present in *A. fumigatus*. These include the cell-surface ferric reductases, ferroxidases (FetC), and the iron permease (FtrA). Like *C. albicans* Ftr1, the *FTRA* gene of *A. fumigatus* is also expressed under iron starvation. Mutants with an inactivated *FTRA* gene showed no difference in growth on iron-depleted medium and in virulence models compared to wild-type *A. fumigatus*, thereby indicating that the permease is not a virulence factor in *A. fumigatus* [74,75]. In Mucorales, this system was shown to be strongly regulated, particularly in low iron conditions [47,48]. Recently, it was demonstrated that there is overexpression of the ferroxidases (*FET3*) in the lung of mice confronted with invasive *M. circinelloides* [47]. In addition, there are three characterised copies of *FET3* (Table 1.) in *M. circinelloides*, which were identified as *FET3A*, *FET3B,* and *FET3C,* with the latter being the most important for infection [47]. Single and double knockout strains of the *FET3* genes were also shown to be critical components involved in iron uptake, particularly in low iron conditions both in vitro and in vivo [47]. In *R. delemar*, the complete deletion of the iron permease (*FTR1*) results in reduced virulence [48,66]. Interestingly, iron starvation induces the metacaspase dependent apoptotic response in strains lacking *FTR1* [48]. In addition, there remains the possibility that the reductive pathway and the iron permeases (Ftr1) in Mucorales may also have a role in scavenging iron from other host proteins, e.g., ferritin or transferrin [40,64,76]. These examples highlight the importance of the reductive pathway has in survival and virulence under iron starvation.

## 3. Haem and Haemoglobin Utilisation

In the host, approximately 60–70% of the iron is bound to haem in haemoglobin, as well as other haem-containing proteins [77,78]. Other host-proteins that bind iron include haemopexin, haptoglobin, lactoferrin, lipocalin-1, and lipocalin-2. Additionally, intracellular iron is bound to ferritin, the second-largest reservoir of iron, and transferrin [78,79,80]. This sequestration of iron to specific proteins prevents the iron-dependent catalysis of free radical cascades and the production of toxic components [2]. Importantly, this mechanism restricts iron availability to invading pathogens [19]. Consequently, the ability of a fungal pathogen to sequester iron from different host iron-containing proteins for growth and proliferation is a key virulence determinant [81]. It has been thoroughly demonstrated that *C. albicans* has a mechanism to obtain iron from haem and haemoglobin that is independent of both the reductive and non-reductive systems, i.e., xenosiderophore transport [82,83]. The ability to utilise haem/haemoglobin depends on the conserved family of common in several fungal extracellular membrane proteins or CFEM proteins, which has an eight cysteine-containing domain: Rbt5, Rbt51/Pga10, Pga7, and the secreted haemophore, Csa2 [81,84,85,86,87,88,89]. The currently accepted model for haeme/haemoglobin uptake suggests that there is a cooperation between Rbt5 and Pga7 [40]. The cell wall-associated Rbt5 facilitates diffusion of haem/haemoglobin across the cell wall and thus accessible to Pga7, which allows internalisation of haem/haemoglobin by endocytosis [81,90]. This model was supported by individual mutants lacking Rbt5 and Pga7, respectively [81,90,91]. Mutants of the latter exhibited significant growth deficiencies in medium containing haem or haemoglobin as the only iron source. In a mouse model of systemic infection, this mutant was attenuated for virulence [90]. Csa2, is another CFEM protein that is also required for *C. albicans* growth on haemoglobin. Structural resolution of the Csa2 protein has identified a novel six α-helix motif with a hydrophobic platform, which may facilitate attachment of planar haem molecules [81,87,90]. This work added to the model for haem-iron transport in *C. albicans*, where haem is cleaved from haemoglobin by Csa2, transferred to the CFEM proteins Rbt5 and Pga7 for internalisation by endocytosis [81,84,85,87,90]. Pinksy et al., 2020 recently demonstrated that *C. albicans* strains lacking *CSA2* and *RBT5* utilise haemoglobin at a weaker rate in comparison to the wild type. Importantly, mutants lacking *PGA7* were unable to utilise haemoglobin as a sole iron source in vitro [91]. The addition of human serum albumin (HSA) with haemoglobin restored growth in *CSA2* mutants similar to the wild type. However, HSA did not rescue growth in *RBT5* and *PGA7* mutants [91]. Growth of the *CSA2* mutants on haemin was similar to the wild type with or without HSA, while mutant *RBT5* strains showed slightly improved growth in the presence of HSA. In contrast, HSA added with haemin completely abolished growth of *PGA7* mutants. However, growth is seen when higher concentrations of haemin are used as the only iron source. These results strongly indicate that the Pga7 protein is an essential member of the CFEM haemophore cascade and it is required for the uptake/utilisation of albumin-bound haemin [91]. Their results also showed that *C. albicans* cannot utilise haem bound to haemopexin (serum haem-binding protein). Adding further to haem utilisation, the expression of only Rbt51 is enough to confer the ability to use haemoglobin in *S. cerevisiae* [81,92]. Mutants of *RBT51* in *C. albicans* grow poorly on either haem or haemoglobin [81,85]. Other pathogenic *Candida* species can utilise haem and haemoglobin to various degrees. For example, *C. auris*, *C. parapsilosis,* and *C. tropicalis* can grow on haem and haemoglobin, but *C. glabrata* and *C. krusei* cannot use these iron sources [76,81,86,91,93,94]. Interestingly, anti-Rbt51 antiserum reacted with lysates from *C. parapsilosis* and *C. tropicalis* but was non-reactive with lysates from *C. glabrata* and *C. krusei*. The inability of *C. glabrata* to exploit haem or haemoglobin suggests that this pathogenic fungus is not well adapted to the host microenvironment, i.e., alkaline pH [76,81,95]. *C. neoformans* can also utilise haem as an iron source [96,97,98,99]. This is facilitated by the endosomal sorting complex required for transport or ESCRT-I protein Vps23, which is involved in haem uptake e.g., by endocytosis [99]. Other proteins involved in haem utilisation include Vps22, and Vps20/Snf7, which are components of the cytosolic protein complexes ESCRT-II and ESCRT-III, respectively [96,98,99]. Recently, Bairwa et al., 2019 confirmed additional proteins involved in the clathrin-mediated endocytosis (CME) of haem/haemoglobin by *C. neoformans* [100]. Their work strongly suggested that the clathrin heavy chain (Chc1) protein (a component of CME), may have a central role in the uptake and trafficking of haem/haemoglobin. This was demonstrated by the impaired ability of strains lacking the *CHC1* gene to internalise haemoglobin. Additionally, *CHC1* mutants were unable to grow in medium containing haemin or haemoglobin as the sole iron source [100]. Furthermore, the loss of *CHC1* abolishes growth at 37 °C, which is a key virulence determinant for *C. neoformans* infection. Other components of the CME pathway involved in haem/haemoglobin utilisation include the Las17 protein, which is the yeast homolog of the Wiskott-Aldrich Syndrome (Wasp) protein in mammals, and the amphiphysin-like lipid raft proteins Rsv161 and Rsv167. Similar to the *CHC1* mutants, strains lacking *LAS17*, *RSV161* and *RSV167* showed impaired growth on haemin containing medium. Mutants of *LAS17* were unable to utilise iron from haem and showed increased survival in preliminary in vivo mouse models. As such, these results indicate that the CME pathway may have an important role in haem utilisation, growth and virulence of *C. neoformans* in vivo [100]. Although haem/haemoglobin utilisation has yet to be demonstrated in Mucorales, putative haem oxygenase genes have been identified in *R. arrhizus* and *L corymbifera* [101,102].

## 4. Siderophore Uptake

**Figure 2:**
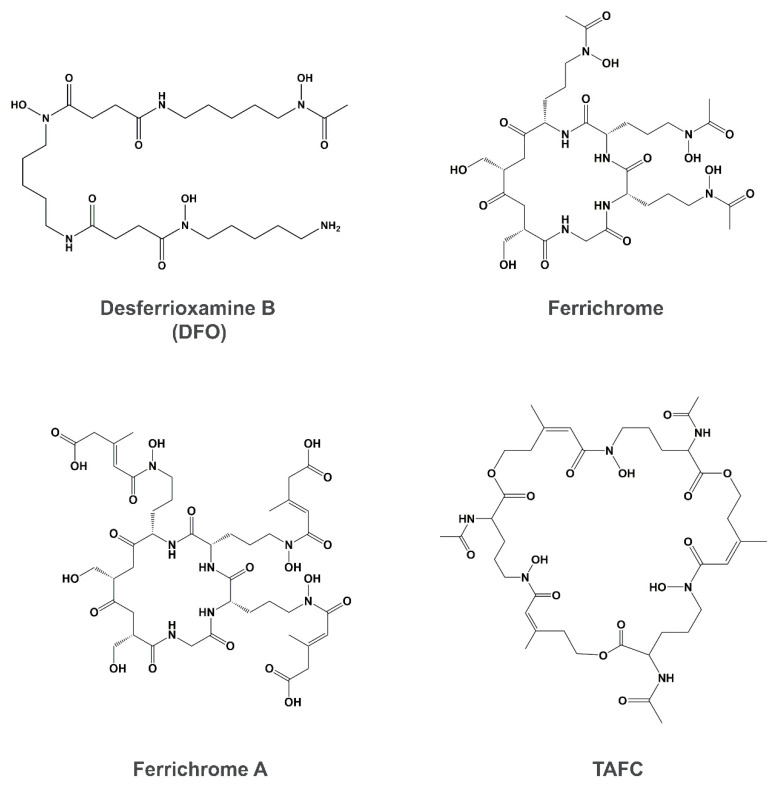

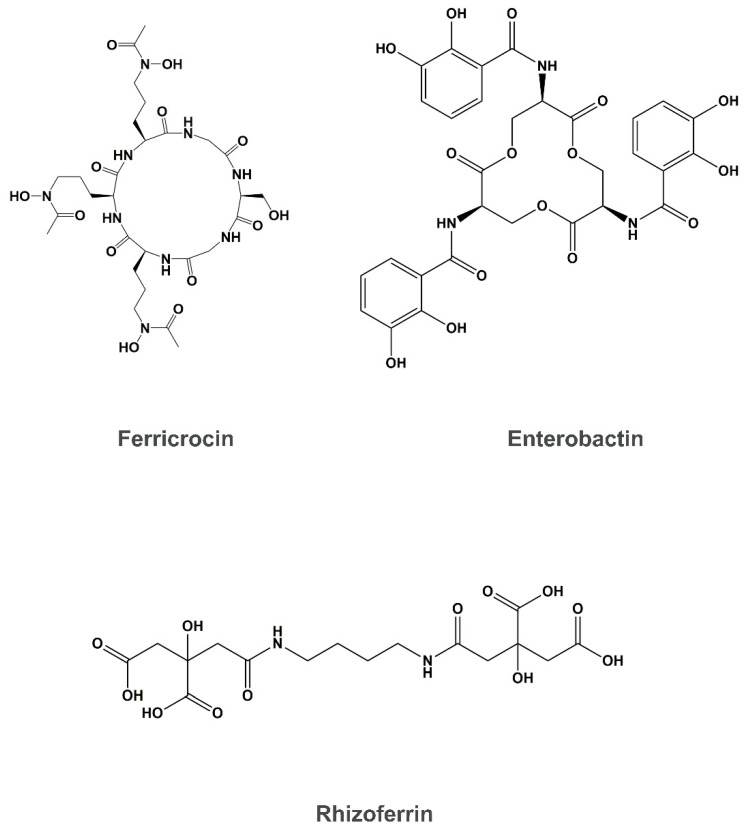
Representative structures of the different classes of siderophores: the Hydroxamate class —desferrioxamine B, ferrichrome, ferrichrome A and TAFC; the Catecholate class—enterobactin; and the Carboxylate—rhizoferrin. All structures are illustrated in their iron-free forms.

Siderophore uptake is a non-reductive iron uptake pathway that contributes to iron acquisition in fungi [40]. Siderophores are small-molecules (usually <1 kDa) that are high-affinity ferric iron chelators secreted by fungi and used as another indirect strategy to sequester iron from all available sources in the environment and in the host [103,104]. In some opportunistic fungal pathogens, i.e., Mucorales, the use of siderophores as therapy directly predisposes to infection [105,106]. Siderophore molecules can be divided into three main classes, depending on the chemical nature of the group donating the oxygen ligands for Fe^3+^; these are the catecholates, hydroxamates, and α-hydroxy carboxylates [107,108]. However, other siderophores containing more complex structures which integrate at least two classes into one molecule, are classified as mixed-type siderophores [108,109]. Representative structures of the three main classes are illustrated in Figure 2. 

Most fungi can synthesise and secrete siderophores that bind ferric iron with extremely high affinity and specificity [110,111,112]. This iron-binding event, specifically for Fe^3+^, has a dissociation constant of approximately 10^−29^ M, significantly greater than other biologically relevant iron-binding ligands in nature [3,112,113]. It is worth noting that the majority of the siderophores synthesised by fungi belong to the hydroxamate class [29,114]. A notable exception is rhizoferrin, a carboxylate-type siderophore that is produced by various Mucorales [29,107,115]. In siderophore-producing organisms, the production of one or more of these compounds is associated with iron starvation (intracellular iron concentration < 10^−6^ M), which has effects on growth [104,111]. Upon secretion, siderophores form stable, extracellular complexes with Fe^3+^. Once iron-bound, the complex can be directly transported by the membrane-bound siderophore-transporters or the entire complex undergoes reduction and oxidation, next the iron (Fe^3+^) is then transported by the Ftr1 of the reductive pathway [113,116]. However, the reductive pathway functions more efficiently in the presence of high concentrations of siderophore-bound iron [3,116]. Numerous fungi express siderophore transporters capable of transporting xenosiderophores, i.e., siderophores produced by other fungal species or bacteria (Table 2) [92,117]. 

**Figure 3:**
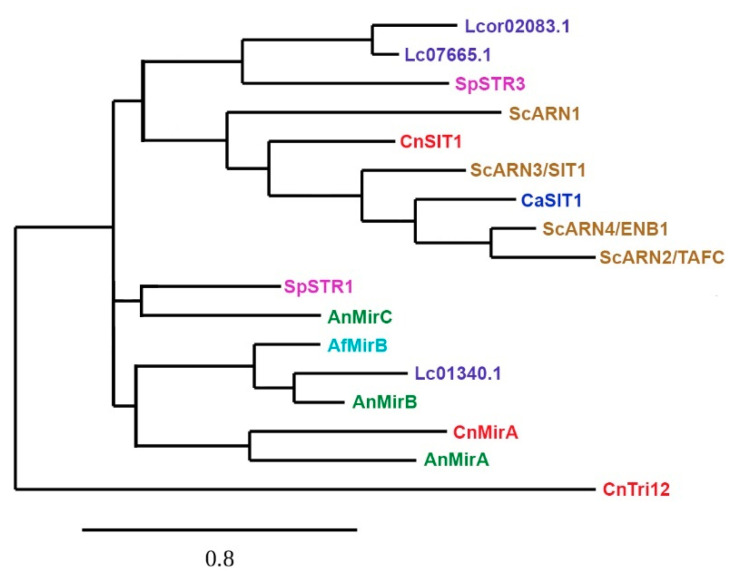
Phylogenetic analysis of characterised siderophore transporters from *S. cerevisiae* (Sc, brown), *S. pombe* (Sp, pink), *C. albicans* (Ca, blue), *C. neoformans* (Cn, red), *A. fumigatus* (Af, torquoise)*,* and *A. nidulans* (An, green). Putative siderophore transporters from *L. corymbifera* (Lc, purple) are also included. CnTri12 is a major facilitator not belonging to the SIT-family of proteins and serves as an outgroup. All sequences were aligned using MUSCLE (v.3.8.31, Marceille, France). Phylogenetic tree was reconstructed using the maximum likelihood method implemented in the PhyML program (v3.1/3/0 aLRT, Marceille, France). The WAG substitution model was selected assuming an estimated proportion of invariant sites (0.011) and 4 gamma-distribution rate categories to account for rate heterogeneity across sites. The gamma shape parameters were estimated directly from the data (gamma = 2.830). Reliability for internal branch was assessed using the aLRT test (SH-Like) [127,128,129,130,131,132,133].

The utilisation of xenosiderophores is advantageous to pathogenic fungi as it means this facilitation, binding, and transport provides better access to iron for growth and proliferation in the host [60,103,113,122,123]. *S. cerevisiae*, *C. albicans*, and *C. neoformans* do not synthesise their own siderophores but can utilise several xenosiderophores produced by other organisms, e.g., ferrichrome [110,124]. Early studies in *S. cerevisiae* provided a clear model for the uptake mechanisms for xenosiderophores via the Arn/Sit transporters belonging to the major facilitator superfamily [3,35,55]. This family of transporters identified as Arn1, Arn2/Taf1, Arn3/Sit1, and Arn4/Enb1, each show specificity for the different classes of siderophores produced by fungi and bacteria [111]. The Arn1 proteins transport ferrichrome, other hydroxamates of the ferrichrome-type, and coprogen [125]. Arn2/Taf1 specifically transports triacetylfusarinine C (TAFC) [118,125]. The Arn3/Sit1 membrane proteins exhibit a broad substrate specificity in comparison to the other transporters, as it recognises a variety of ferrichromes, coprogen as well as bacterially derived ferrioxamines [3,29,35,55,93,111]. Arn4/Enb1 exclusively transports the catecholate siderophore, Enterobactin produced by *E. coli* [65,126]. This phylogenetic relationship between the characterised and putative siderophore transporter genes are illustrated in (Figure 3).

The Arn transporters are internalised when the ligands are bound to a siderophore [111]. This complex is then transported via late endosomal vesicles for vacuolar degradation which releases the iron [119]. This pathway was elegantly shown by Yun et al., 2001 where the trafficking of ferrichrome and ferroxamine B are transported by Arn3/Sit1 and Arn1 transporters, respectively [35,119]. The transcriptional activator Aft1 in *S. cerevisiae* responds to iron availability and regulates the expression of *FET3, FTR1,* and *ARN*; it has also been shown to interact with Arn3/Sit1 transporters [119]. This interaction influences the ubiquitination and vacuole-dependent degradation of the protein, further illustrating that the sensing mechanisms in fungi can be adjusted accordingly for efficient iron uptake [134,135]. In *C. albicans*, the Arn1/Sit1 transporter is used to facilitate the utilisation of xenosiderophores such as coprogen, ferrichrysin ferricrocin, ferrirubin, and triacetyl-fusarine C [93,125]. As a human commensal, *C. albicans* share the mucosal and gastrointestinal environment with other flora including bacteria, thus the ability to utilise xenosiderophores was most likely developed in this environment [125,136]. The Arn1/Sit1 transporter in *C. albicans* was shown to be required for the invasion of reconstituted epithelium in a human oral mucosa model [125]. However in mouse models of systemic candidiasis, it does not contribute to virulence, thus indicating that siderophore-mediated iron uptake may not be important during bloodstream infections [125]. For *C. glabrata*, it was demonstrated that *SIT1* mutants showed no difference in survival within the phagolysosome of infected macrophages when compared to the wild-type strain [93,137]. The highly conserved *SIT1* transporter of *C. neoformans* is not involved in virulence in murine models of cryptococcosis, but it plays a role in the uptake of ferrioxamine B as well as other environmental xenosiderophores [138]. In addition, the Cft1 and Cfo1 of the reductive pathway is not required for iron assimilation from ferrioxamine [139]. Xenosiderophores are of high importance in mucormycosis caused by the Mucorales, i.e., *Rhizopus, Mucor, Lichtheimia,* as well as uncommon genera, e.g., *Apophysomyces* [42,140]. The most common causative agents isolated in nosocomial infections include *R. arrhizus* and *L. corymbifera* [140,141]. Mucormycosis is seen in immunocompromised patients, poorly managed diabetic patients, diabetics with ketoacidosis experiencing iron overload (DKA), and patients in end-stage renal failure on chelation therapy, e.g., desferoxamine B siderophore treatment [141,142,143]. *R. arrhizus* and other pathogenic Mucorales possess cell surface-proteins, i.e., Fob1 and Fob2, which allows for the exploitation of desferoxamine thus aiding growth in the host [71,115,142,144]. In addition, the reductive iron uptake pathway also provides an additional mechanism for siderophore-Fe^3+^ utilisation, and contributes to pathogenesis as defects in this pathway, i.e., mutants of the iron permease (*FTR1*) show attenuated virulence [71,72,101,145]. 

In *A. fumigatus* and *A. nidulans* as well as in *Histoplasma capsulatum*, siderophore-mediated iron uptake has been thoroughly studied as knockout strains can be obtained [29,34,38,124,146,147]. *Aspergillus* species and *H. capsulatum* can synthesise different hydroxamate-type siderophore. *Aspergillus* species can synthesise fusarinine C, triacetylfusarinine C, ferricrocin, hydroxferricrocin, while *H. capsulatum* produces coprogen B [29,146,148,149,150,151]. In *A. fumigatus*, the *SIDA* gene that encodes the L-ornithine-N-monooxygenase is essential for siderophore production and contributes to virulence [75,147,152]. *A. fumigatus* also possess a highly conserved orthologs of the *S. cerevisiae SIT1* and *SIT2* transporter genes, and it was demonstrated that these transporters play a role in the uptake of ferrioxamine B and ferrichrome [153]. The deletion of the *SID1* gene (ortholog of *SIDA*) in *H. capsulatum*, abolishes the siderophore biosynthesis pathway and inhibits fungal growth in bone marrow-derived macrophages and in mice. This indicates that siderophore production is an essential virulence mechanism for *H. capsulatum* [154]. The siderophore uptake system enables fungi to effectively compete for limited amounts of available iron in the environment and allows opportunistic fungi another mechanism to efficiently acquire iron during infection. 

## 5. The Fungal Cell Wall: Composition and Role in Diagnostics

### 5.1. The Cell Wall Composition of Mucorales in Comparison to Other Fungi

**Figure 4:**
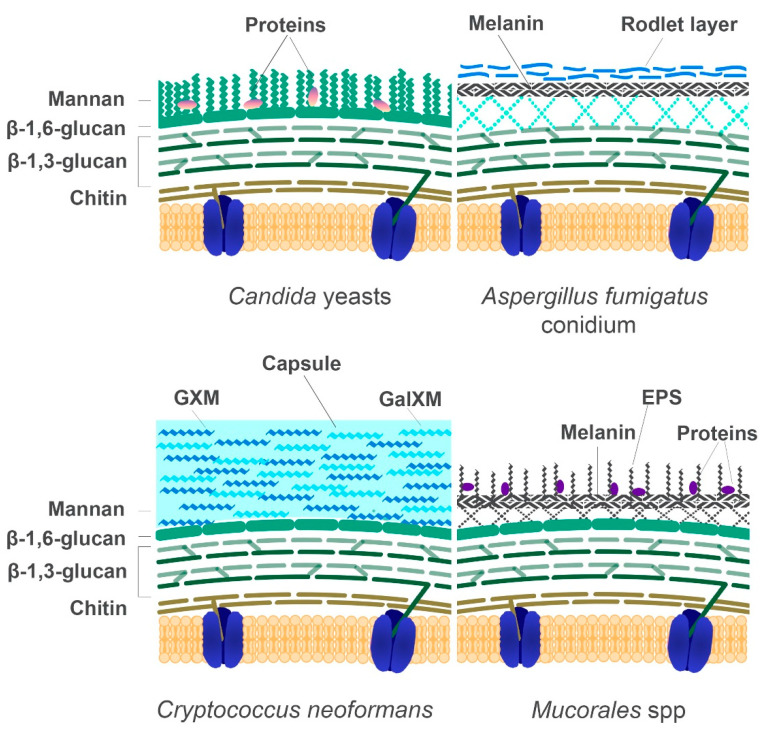
Schematic organisation of the fungal cell wall of opportunistic fungi. This illustration shows the major components of the cell wall based on current knowledge of the fungal model. Most fungi have chitin, branched β-1,3-glucan and β-1,6-glucan with notable differences in their architecture and attachments to these basal components. In the yeast, *C. albicans*, there is an inner layer of chitin, followed by a β-1,3- glucan and β-1,6-glucan foundation that anchors glycosylphosphatidylinositol-linked (GPI) glycoproteins. In the conidia of *A. fumigatus*, the basal layer consists of β-1,3- and β-1,4-glucans which are attached to a linear α-1,3 and α-1,6-glucan layer. The mannan chains in *A. fumigatus* are low molecular weight β-1,5-galactofurans. The cell wall of *A. fumigatus* conidia possesses a hydrophobic layer known as the hydrophobin rodlet layer and a melanin layer; the hyphae consists of α-1,3-glucans, galactomannan (GM), galactosaminoglycan (GAG) and a few glycosylated proteins (no illustrated). The cell wall of the Basidiomycetous yeast *C. neoformans*, consists of a β-1,3-glucan and β-1,6-glucan foundation, a mixture of chitin/chitosan. This is followed by the α-1,3 glucans anchor, the capsule outer layer which consists of glucuronoxylomannan (GXM) and galactoxylomannan (GalXM). The precise structure of the *Mucorales* cell wall is yet to be fully characterised for both the sporangiospores and hyphal form. Illustrated here is the partially known component of the Mucorales sporangiospore. To date, the cell wall has been shown to consist of chitin/chitosan, β-1,3-glucans, mannan, mannose, extracellular polysaccharides (EPS) and other polysaccharides, e.g., mucoran and mucoric acid (hyphae); figure adapted from [170,171].

The cell wall is an essential structure that maintains the integrity and viability of the fungal conidia, protecting the cell from the harsh external environment [3,155]. It also confers the distinct and identifiable morphology that houses important antigenic determinants which are vital for adhesion, receptor-mediated signalling cascades within the conidia, and subsequent colonisation leading to disease [61,156]. For nutrients, e.g., iron in any form or iron containing compounds, to gain access to the plasma-membrane and the embedded uptake-systems, these compounds must first traverse the fungal cell wall and cross the periplasmic space (Figure 1.) [3,61,157]. As such, the fungal cell wall must have some level of regulated permeability [157,158]. The intricate structure of the cell wall consists of a meshwork of complex β-1,3-glucans, chitin, an outer layer of mannoproteins as well as lipids, glycoproteins, and pigments (Figure 4) [55,61,159,160,161,162]. These components are highly immunogenic and elicit both cellular and humoral response during infection [159,163,164,165]. Interestingly, the mannoprotein composition and the permeability of the cell wall changes under different growth stages and conditions, e.g., exposure to antifungals [61,158,160,166]. This subsequently alters the passage of nutrients through the cell wall into the periplasmic space and to the plasma membrane. As such, the fungal cell wall represents an ideal target for antifungals [158,167,168,169].

Fungal β-glucans, which represents approximately 50–60% of the structure’s dry mass, are the most abundant polysaccharides in the cell wall and are characterised by the presence of β-(1,3-)-glucans backbone with or without branches of β-(1,6-)-linked glucans, β-(1,4-), α-1,3 and α-1,4 links [172]. For example, the cell wall of *C. albicans* contains β-(1,6-) linkages, while *Aspergillus* spp. do not [165,168,173,174]. The most important component of the cell wall is the β-1,3-D-glucan, which is synthesised by 1,3-β-D-glucan synthase. This protein complex consists of two subunits: (1) Fks1, which is the catalytic subunit that produces the glycosidic bonds, and (2) Rho1, which is a Ras-like GTP-binding protein that regulates the activity of the β-D-glucan synthase [175,176,177,178]. Chitin, on the other hand, accounts for 1-2% of the cell wall content and is made up of a linear polymer of β-1,4-linked acetylglucosamine (β-1,4-linked GlcNAc), which forms microfibrils in the cell wall [160,179,180]. The synthesis of chitin from N-acetylglucosamine is catalysed by the chitin synthase enzyme, afterwards, the chitin polymers are deposited on the outer region of the plasma membrane [179,181,182,183,184]. The glycoproteins represent between 30–50% of the dry mass of the fungal cell wall, e.g., in *S. cerevisiae* or *Candida* spp. They are composed of modified N-and O-linked carbohydrates or mannan [168]. In some fungi, the mannan backbone consists of either single residues or side chains of different sugars [180,185]. These glycoproteins have diverse functions, from participating in maintenance and remodelling of the cell wall structure to adhesion and in signal transduction into the cytoplasm [157,168,180,186]. Another component of the cell wall that has been shown to be important for protection, survival, and viability of the conidia is the pigment melanin. This pigment has a relatively high molecular weight that is negatively charged, hydrophobic, and insoluble [162,187,188,189]. Melanin plays an important role in fungal virulence for some pathogenic fungi, as it has a role in the inhibition of phagocytosis as well as disturbing host immune response, invasion, and dissemination [162,184,188,190,191,192,193]. The presence of melanin offers protection from oxidative stresses, temperature, and UV damage [159,161,191,192]. Overall, these various components of the fungal cell wall represents ideal targets for diagnosis and antifungals treatment [158,167,168,169]. 

### 5.2. Diagnostic Methods Based on Properties of the Fungal Cell

Numerous challenges arise for the accurate diagnosis of invasive fungal infections (IFIs) in immunocompromised patients, especially those with underlying malignancies and/or HSCT [194]. The clinical manifestations are non-specific, usually requiring a degree of suspicion for early recognition and prompt antifungal treatment [195]. According to the International Society for Heart and Lung Transplantation, IFIs are defined as the presence of fungus in the respiratory secretions including sputum or bronchoalveolar lavage (BL) detected by PCR, biomarkers or cultures in the presence of symptoms, radiological and endobronchial changes or signs of histological changes indicative of tissue invasion by fungi [195,196,197]. The invasive fungal infections cooperative group (IFICG) of the European Organisation for Research and Treatment for Cancer (EORTC) and Mycology Study Group (MSG) of the National Institute of Allergy and Infectious Diseases (NIAID) have published standard classifications of IFIs for research purposes. These classifications apply possible, probable and proven to the patient evaluation data based on a combination of host factors, clinical presentations, microbiological and biomarker indications [195,197,198]. The standard diagnostic tools used in the clinical setting to diagnose IFIs are summarised in Table 3. To date, more comprehensive reviews are available that provide details on the most relevant and applicable diagnostic techniques currently used in the clinical settings [199,200,201,202,203,204].

## 6. Iron Acquisition and Susceptibility to Antifungals: Implications in Therapy

### 6.1. Antifungal Treatment and Iron Chelation Therapy

Successful management of IFIs are based on the timely initiation of optimal antifungal therapy, reversal or discontinuation of underlying predisposing factors and the use of relevant adjunctive therapies [222]. Additionally, immediate correction of metabolic disorders or abnormalities in patients with uncontrolled diabetes is mandatory in suspected mucormycosis cases. Surgical intervention for the complete removal of infected tissue in urgent cases significantly improves patient outcome [199,202,204,223]. Only four classes of antifungal medications are currently available for the treatment of IFIs, these are: polyenes, pyrimidine analogue, echinocandins and triazoles [207,210,224]. The latter i.e., echinocandins and azoles will be discussed later as emerging resistance is becoming more prevalent [225]. The first line treatment of invasive candidiasis is typically the echinocandins as well as formulations of amphotericin B (AMB) [205]. For Cryptococcal infections, the gold standard antifungal drugs include the polyenes, flucytosine (5-FC), triazoles and their combinations [226,227]. Treatment options for invasive aspergillosis include voriconazole, liposomal amphotericin B (LAMB) and most recently isavuconazole [199,228,229]. In mucormycosis, the lipid formulations of AMB, i.e., LAMB and AMB lipid complex, (AMLC) is the optimal treatment option [199,202,204,222,228,230]. It is important to note that Mucoralean fungi are innately resistant to most antifungals in vitro, including voriconazole [231]. Most recently, posaconazole and isavuconazole have exhibited activity against Mucorales [228,231,232].

Currently, therapeutic strategies to combat complicated infections as well as innate, emerging resistance in fungal pathogens include adjunctive therapies and new antifungal drugs [233]. Adjunctive therapies functions by interfering with resistance mechanisms or modifying drug activity [197]. Examples of the former include efflux pump inhibitors, which increase intracellular antifungal concentration, and histone deacetylase inhibitors, which are used in combination with azoles to inhibit fungal growth [197,234,235,236]. Compounds that modify antifungal activity usually act synergistically by altering the fungal stress response mechanisms [237,238,239,240]. These include statins, heat-shock protein 90 (Hsp90) inhibitors, nonsteroidal anti-inflammatory drugs, inhibitors of calcineurin and calmodulin, calcium homeostasis, selective serotonin reuptake, and iron homeostasis [197,207]. 

Iron metabolism holds a central role in fungal pathogenesis, particularly in the development of mucormycosis. Thus, there is the possibility to use iron chelators as an adjunctive therapy strategy as this could limit/inhibit fungal growth. The iron chelator deferasirox is used for the treatment of iron overload in immunocompromised patients and those with elevated serum iron, e.g., diabetic & DKA patients. Preclinical data on DKA murine models of *R. oryzae (R. arrhizus)* infection found that treatment with deferasirox was as effective as LAMB therapy and combination treatment, i.e., deferasirox-LAMB, acted synergistically to improve survival [31,101,143,241,242]. Although this showed promise, in the clinical application of deferasirox-LAMB, it was demonstrated to significantly increase mortality in patients with hematologic malignancies [101,210,241,243,244]. On the other hand, this treatment strategy remains a viable option for other high-risk patient groups, e.g., DKA patients [200,202]. Deferasirox was also seen to enhance LAMP treatment in a murine model of invasive pulmonary aspergillosis. However, relevant clinical applications or data remain lacking [242,245]. Synergy was shown with fluconazole, ketoconazole, or AMB when combined with other iron chelators, including deferiprone, lactoferrin, and ciclopirox. These combinations proved successful in inhibiting *A. fumigatus* growth in vitro [237]. Another potential novel target for the treatment of Mucorales include the inhibition or blocking of the proteins involved in the reductive pathway. Antibodies targeting the iron permeases (Ftr1) of *R. oryzae (R. arrhizus)* protected DKA mice from infection [33,72,246]. Additionally, antibodies targeting the unique host proteins involved in receptor mediated endocytosis of fungal spores, i.e., 78kDa glucose-regulated protein (Grp78/HspA5) are possible targets. Grp78/HspA5 is overexpressed in patients with hyperglycaemia, DKA, and elevated serum iron; thus, antibodies, i.e., anti-Grp78 may be promising novel targets as it was shown to offer protection in a murine DKA model. Similar protective attributes were seen when antibodies of the fungal spore coat protein H or CotH i.e., anti-CotH (the interaction partner of Grp78/HspA5) were used in DKA murine model [105,231,247]. 

### 6.2. Antifungal Resistance and Iron

#### 6.2.1. Echinocandins 

**Figure 5:**
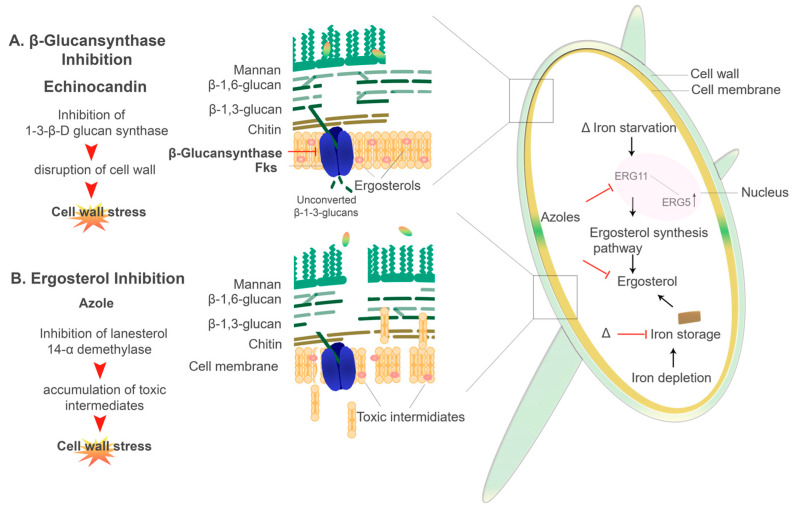
Azoles and echinocandin antifungal drugs and their mechanism of actions: An illustration of two main classes of antifungal drugs used clinically and how they affect the fungal cell of *C. albicans*. (**A**) Echinocandins, e.g., caspofungin, inhibit β-(1-3)-D-glucan synthase in the cell membrane, which leads to disruption in cell wall integrity. (**B**) Azoles, e.g., fluconazole, inhibit Erg11/CYP51 F5, which blocks the production of ergosterol, leading to the accumulation of toxic sterol intermediates. **Δ** indicates where iron starvation or depletion may contribute to increased susceptibility to azole antifungals.

Antifungal compounds that specifically target the cell wall components include Ibrexafungerp (SCY-078) and the Echinocandins, e.g., caspofungin, micafungin, and anidulafungin [181,248]. Ibrexafungerp (SCY-078) functions by actively inhibiting the 1,3-β-D-glucan synthase while the Echinocandins inhibit the 1,3-β-D-glucan synthase by noncompetitively binding to the Fksp subunit of the enzyme, which leads to a decrease in the amount of β-D-glucans present in the cell wall (Figure 5) [249,250,251,252,253,254]. Cell death is seen in *C. albicans* when this enzyme is inhibited by caspofungin and micafungin [252,254,255,256,257]. Interestingly, Δ*CCC2* cells (defectives in copper transport) show hypersensitivity to echinocandins [258]. On the other hand, elevated proportions of chitin in the cell wall of *Candida* species exhibit increased resistance to caspofungin, particularly in in vivo candidiasis models [238,248,251,259]. Recently, Pradhan et al. 2019 demonstrated that iron-limitations induces a β-glucan masking phenotype as well as cell wall remodelling and thickening. However, defects in this phenotype was observed in mutants lacking the permease and transcription factor (Δ*FTR1* and Δ*SEF1,* respectively) [64]. Through this β-glucan masking, there is reduced phagocytosis and a dramatic reduction in proinflammatory cytokines (TNF-α and IL-6) produced by peripheral blood mononuclear cells (PBMCs) [64,163,164]. However, the use of caspofungin enhances β-glucan exposure [163,164]. Interestingly, the 1-3-β-d-Glucan inhibitor ibrexafungerp appears to be effective against clinical isolates that are resistant to echinocandins [260,261]. The dynamic nature of the cell wall has a major role in the development of antifungal resistance [262,263]. In both *C. albicans* and *A. fumigatus*, changes in the structural composition of the cell wall have been noted in strains showing antifungal resistance [210,213,214].

#### 6.2.2. Azoles 

Azole antifungals have been in clinical use for more than 20 years [264]. The azoles are separated into two distinctive classes, i.e., the triazoles and the imidazoles. Triazoles used in the clinical setting include fluconazole, itraconazole, voriconazole and posaconazole [265]. Common imidazoles used are clotrimazole, ketoconazole and miconazole [265,266]. Cytochrome P450 (CYP450) is an enzyme that converts lanosterol to ergosterol, which is the major sterol in the fungal plasma membrane. Azoles inhibit the CYP450 enzymes which causes increase permeability of the fungal plasma membrane (Figure 5.) [265,266,267]. Azoles also affect other efflux transporters, including major facilitator superfamily (MFS) transporters and ATP-binding cassette (ABC) transporters [268]. Susceptibility to azole antifungals is seen in *Candida* spp., *C. neoformans*, *Aspergillus* spp., and the Mucorales, to name a few. However, resistance has also been well characterised among this class of antifungal therapy [239,268,269,270,271]. The direct target of fluconazole is Erg11 (homologous to the yeast CYP51 F5), an enzyme involved in the ergosterol biosynthesis pathway [97,240,272,273]. 

In *C. albicans*, it was shown that intracellular iron depletion leads to increased fluidity of the plasma membrane as there is reduced ergosterol [240,274]. Gene expression of *ERG11*, which encodes for lanosterol 14-α demethylase as well as the *ERG3* gene, which encodes for the Δ5,6-desaturase is affected by intracellular iron availability (Figure 5.). Erg3 catalyses the addition of a carbon-carbon double bond to the substrate molecules in the finals steps of the ergosterol biosynthesis pathway [39,240,271,275]. The strains lacking the high-affinity iron permease Ftr1 (Δ*FTR1*), null mutants (lacking both: Δ*FTR1* and Δ*FTR2*) as well as *ΔCCC2* mutants (copper transporter) were all shown to be more susceptible to fluconazole [240]. An important note is that the Ccc2 copper transporter is responsible for the copper acquisition, as copper is a key component of the multicopper oxidase (Fet3) protein in the reductive pathway [240,258]. Iron deprivation results in the downregulation of *ERG11* [240,271]. As such, the increased membrane fluidity due to lower ergosterol content seen in the iron uptake mutants (Δ*FTR1*, Δ*FTR2*, Δ*FTR1* Δ*FTR2*, and *ΔCCC2*) leads to higher passive diffusion of azole antifungals, thus increased susceptibility [271,276]. This is compounded by the upregulation of *ERG3*, which in an azole-inhibited pathway, allows for the accumulation of toxic intermediates [39,271]. Therefore, Erg3 acts synergistically with azoles increasing susceptibility [239,240,271]. On the other hand, mutations or deletions of the *ERG3* gene, as well as upregulation of *ERG11,* confers azole resistance in *C. albicans* (Figure 5.) [271,275,277]. Similarly, the reductive iron uptake system in *C. neoformans* has an important role in resistance to azoles [97,138,278]. Mutants lacking both the multicopper ferroxidase (*CFO1*) and the iron permease (*CFT1*) had reduced intracellular iron levels, which significantly increase azole drug susceptibility, i.e., to fluconazole [97,279]. Interestingly, overexpression of *ERG11* in *CFO1* mutants exhibited reduced susceptibility to fluconazole [97,272,277,280]. Innate and acquired reduced susceptibility and resistance to azole in *A. fumigatus* has been linked to numerous point mutations in the *CYP51A* gene [268,270,281]. It has been demonstrated that the Mucorales have an intrinsic resistance to azole antifungals, specifically to the short-tailed azoles, i.e., fluconazole and voriconazole [270,281,282,283,284]. It was found that this intrinsic resistance may be caused by an amino acid substitution in the cytochrome P51 or CYP51 F5 (Erg11) enzyme; changing a Tryosine (Y) to Phenylalanine (F) at position 129 i.e., Y129F [270]. Interestingly, the CYP51 enzyme was shown to be highly regulated by iron in *A. fumigatus* [268,279]. 

## 7. Conclusions

Iron is an absolute requirement for most organisms and biological processes. The information discussed above highlights the complexity of iron assimilation, iron regulation, and homeostasis in fungi. Considering the importance iron has in growth, survival, and virulence, it is not surprising that these intricate mechanisms and pathways also play a role in the development of resistance to antifungal drug therapies. The convolute relationship between iron availability, transport proteins in the fungal cell wall, and membrane components suggest numerous possibilities for new strategies in the treatment of opportunistic fungal pathogens. However, much is yet to be elucidated about the cell wall composition and the iron acquisition pathways in the basal fungal lineage order, Mucorales, with their known resistance to antifungals drug therapies. Given the central role of iron in pathogenesis, combined treatment of antifungals with compounds targeting iron assimilation is a promising approach to combat opportunistic fungal infections, particularly mucormycosis. 

## Figures and Tables

**Table 1:** Reductive iron acquisition system in *Saccharomyces cerevisiae* and pathogenic Mucoralean species.

Component	Species	Gene	Functions	Ref
Ferric reductases	*Saccharomyces cerevisiae*	*FRE1, FRE2*	Ferric iron reduction at the cell surface	[3,36,65]
	*Rhizopus* spp.	*FRE* (homolog)	Putative protein—ferric iron reduction at the cell surface	[48,66]
	*Mucor circinelloides*	*FRE* (homolog)	Putative protein—ferric iron reduction at the cell surface	[47,67]
	*Lichtheimia corymbifera*	*FRE5* (homolog)–three copies	Putative protein—ferric iron reduction at the cell surface	[49]
Multicopper ferroxidase	*S. cerevisiae*	*FET3*	Multicopper-oxidase Ferrous iron oxidation and high-affinity uptake coupled with Ftr1 (permease)	[3,55,65,68]
	*Rhizopus* spp.	*FET3* homolog	Putative multicopper oxidase	[48]
	*M. circinelloides*	*FETA, FETB, FETC*	Ferrous iron oxidation and high-affinity iron uptake	[47]
	*L. corymbifera*	*FET3/5* homolog–three copies	Putative multicopper oxidase	[49]
Iron permease	*S. cerevisiae*	*FTR1*	High-affinity iron uptake, coupled with FET3 (multicopper oxidase)	[3,59,68,69,70]
	*Rhizopus* spp.	*FTR1*	High affinity iron permease	[65,71,72]
	*M. circinelloides*	*FTR1* (homolog)	Putative iron permease	[47,73]
	*L. corymbifera*	*FTR1* (homolog)*—four copies*	Putative iron permease	[49]

**Table 2:** Components of the siderophore transport system in *S. cerevisiae* and in pathogenic Mucoralean species.

Organism	Transporter	Function	Siderophore Substrate	Publication
*S. cerevisiae*	Arn1	Ferrichrome and Ferrichrome A transporter	Ferrichrome and Ferrichrome A	[3,103,118,119,120]
	Arn2/Taf1p	Triacetylfusarinine C (TAFC) transporter	TAFC	[3,118,119]
	Arn3/Sit1p	Ferrichrome and Ferrichrome A transporter	Ferrioxamine B, Ferrichrome A, Ferrichromes, Ferricrocin, Ferrichrycin, Ferrirhodin and Ferrirubin	[3,118,119]
	Arn4p/Enb1p	Enterobactin transporter	Enterobactin	[3,118,119,121]
*R.arrhizus* (*syn. R. oryzae, R. delemar*)	Fob1, Fob2	Ferrioxamine binding	Ferrioxamine B	[48,71]
*L. corymbifera*	Fob1 (putative protein)	Ferrioxamine binding	Ferrioxamine B	[49]

**Table 3:** Techniques used for diagnosis of fungal infections.

	Method	Organism	Comment	Publications
Microscopy	Direct histology and cytology	*Candida* spp.; *Cryptococcus* spp.; *Aspergillus* spp.; Mucorales	Gold standard, demonstration of tissue invasion	[199,205]
Cultures	Mycological culture	*Cryptococcus* spp. *Candida* spp.; *Aspergillus* spp.; Mucorales	Slow turn-around time	[206,207,208,209,210]
	Blood cultures	*Candida* spp.; *A*. *fumigatus,* *A. terreus;*	Gold standard for candidemia;	[211,212]
Serological methods	1,3-β-D-glucan (BDG) *	*Candida* spp.; *Aspergillus* spp.	Exceptions: Mucorales and *Cryptococcus* spp.	[195,197,199,205,213,214,215]
	Galactomannan (GM) enzyme immunoassay *	*Aspergillus* spp.		[216]
Molecular approaches	PCR (18s rDNA, 28s rDNA, ITS, mtDNA	*Candida* spp.; *Cryptococcus* spp.; *Aspergillus* spp.; Mucorales	-	[217,218,219,220]
Imaging technologies	X-rays, CT and CTPA	*Aspergillus* spp.; Mucorales	-	[218,221]
	MRI and PET scan	*Cryptococcus* spp.; *Aspergillus* spp.; Mucorales	-	

* Fungal cell wall component; PCR: polymerase chain reaction; ITS: internal transcribed spacer region; mtDNA: mitochondrial DNA; CT: computerised tomography; CTPA: CT pulmonary angiography; MRI: magnetic resonance imaging; PET: positron emission tomography.

## References

[1] Domenico I., Ward D.M., Kaplan J. (2008). Regulation of iron acquisition and storage: Consequences for iron-linked disorders. Nat. Rev. Mol. Cell Biol..

[2] Cassat J.E., Skaar E.P. (2013). Iron in Infection and Immunity. Cell Host Microbe.

[3] Philpott C.C. (2006). Iron uptake in fungi: A system for every source. Biochim. Biophys. Acta-Mol. Cell Res..

[4] Boyce K.J., Andrianopoulos A. (2015). Fungal dimorphism: The switch from hyphae to yeast is a specialized morphogenetic adaptation allowing colonization of a host. FEMS Microbiol. Rev..

[5] Beard J.L. (2001). Iron Biology in Immune Function, Muscle Metabolism and Neuronal Functioning. J. Nutr..

[6] Krewulak K.D., Vogel H.J. (2008). Structural biology of bacterial iron uptake. Biochim. Biophys. Acta-Biomembr..

[7] Rouault T.A., Tong W.H. (2008). Iron–sulfur cluster biogenesis and human disease. Trends Genet..

[8] Prousek J. (2007). Fenton chemistry in biology and medicine. Pure Appl. Chem..

[9] Halliwell B., Gutteridget J.M.C. (1984). Oxygen toxicity, oxygen radicals, transition metals and disease. Biochem. J..

[10] Brault A., Mourer T., Labbé S. (2015). Molecular basis of the regulation of iron homeostasis in fission and filamentous yeasts. IUBMB Life.

[11] Kaplan J., McVey Ward D., Crisp R.J., Philpott C.C. (2006). Iron-dependent metabolic remodeling in *S. cerevisiae*. Biochim. Biophys. Acta-Mol. Cell Res..

[12] Miethke M. (2013). Molecular strategies of microbial iron assimilation: From high-affinity complexes to cofactor assembly systems. Metallomics.

[13] Carrano C.J., Böhnke R., Matzanke B.F. (1996). Fungal ferritins: The ferritin from mycelia of *Absidia spinosa* is a bacterioferritin. FEBS Lett..

[14] Ibrahim A.S., Spellberg B., Walsh T.J., Kontoyiannis D.P. (2012). Pathogenesis of mucormycosis. Clin. Infect. Dis..

[15] Comensoli L., Bindschedler S., Junier P., Joseph E. (2017). Iron and Fungal Physiology: A Review of Biotechnological Opportunities. Adv. Appl. Microbiol..

[16] Doguer C., Ha J.-H., Collins J.F. (2018). Intersection of Iron and Copper Metabolism in the Mammalian Intestine and Liver. Comprehensive Physiology.

[17] Kehl-Fie T.E., Skaar E.P. (2010). Nutritional immunity beyond iron: A role for manganese and zinc. Curr. Opin. Chem. Biol..

[18] Lane D.J.R., Merlot A.M., Huang M.L.H., Bae D.H., Jansson P.J., Sahni S., Kalinowski D.S., Richardson D.R. (2015). Cellular iron uptake, trafficking and metabolism: Key molecules and mechanisms and their roles in disease. Biochim. Biophys. Acta-Mol. Cell Res..

[19] Hood M.I., Skaar E.P. (2012). Nutritional immunity: Transition metals at the pathogen–host interface. Nat. Rev. Microbiol..

[20] Pfaller M.A., Diekema D.J. (2010). Epidemiology of invasive mycoses in North America. Crit. Rev. Microbiol..

[21] Sam Q.H., Yew W.S., Seneviratne C.J., Chang M.W., Chai L.Y.A. (2018). Immunomodulation as Therapy for Fungal Infection: Are We Closer?. Front. Microbiol..

[22] Rodrigues M.L., Nosanchuk J.D. (2020). Fungal diseases as neglected pathogens: A wake-up call to public health officials. PLoS Negl. Trop. Dis..

[23] Low C.Y., Rotstein C. (2011). Emerging fungal infections in immunocompromised patients. F1000 Med. Rep..

[24] Jabra-Rizk M.A., Kong E.F., Tsui C., Nguyen M.H., Clancy C.J., Fidel P.L., Noverr M. (2016). Candida albicans Pathogenesis: Fitting within the Host-Microbe Damage Response Framework. Infect. Immun..

[25] Armstrong-James D., Meintjes G., Brown G.D. (2014). A neglected epidemic: Fungal infections in HIV/AIDS. Trends Microbiol..

[26] Warkentien T., Crum-Cianflone N.F. (2010). An update on *Cryptococcus* among HIV-infected patients. Int. J. STD AIDS.

[27] Rajasingham R., Smith R.M., Park B.J., Jarvis J.N., Govender N.P., Chiller T.M., Denning D.W., Loyse A., Boulware D.R. (2017). Global burden of disease of HIV-associated cryptococcal meningitis: An updated analysis. Lancet Infect. Dis..

[28] Franco-Paredes C., Chastain D.B., Rodriguez-Morales A.J., Marcos L.A. (2017). Cryptococcal meningoencephalitis in HIV/AIDS: When to start antiretroviral therapy?. Ann. Clin. Microbiol. Antimicrob..

[29] Haas H. (2014). Fungal siderophore metabolism with a focus on *Aspergillus fumigatus*. Nat. Prod. Rep..

[30] Wiederhold N.P. (2017). Antifungal resistance: Current trends and future strategies to combat. Infect. Drug Resist..

[31] Ibrahim A.S., Spellberg B., Edwards J. (2008). Iron acquisition: A novel perspective on mucormycosis pathogenesis and treatment. Curr. Opin. Infect. Dis..

[32] Symeonidis A.S. (2009). The role of iron and iron chelators in zygomycosis. Clin. Microbiol. Infect..

[33] Ibrahim A.S. (2014). Host-iron assimilation: Pathogenesis and novel therapies of mucormycosis. Mycoses.

[34] Haas H. (2012). Iron—A key nexus in the virulence of *Aspergillus fumigatus*. Front. Microbiol..

[35] Yun C., Ferea T., Rashford J., Ardon O., Brown P.O., Botstein D., Kaplan J., Philpott C.C. (2000). Desferrioxamine-mediated Iron Uptake in *Saccharomyces cerevisiae*. J. Biol. Chem..

[36] Baker Brachmann C., Davies A., Cost G.J., Caputo E., Li J., Hieter P., Boeke J.D. (1998). Designer deletion strains derived from *Saccharomyces cerevisiae* S288C: A useful set of strains and plasmids for PCR-mediated gene disruption and other applications. Yeast.

[37] Hassett R.F., Romeo A.M., Kosman D.J. (1998). Regulation of High Affinity Iron Uptake in the Yeast *Saccharomyces cerevisiae* ROLE OF DIOXYGEN AND Fe (II). J. Biol. Chem..

[38] Leal S.M., Roy S., Vareechon C., Carrion S., deJesus J., Clark H., Lopez-Berges M.S., DiPietro A., Schrettl M., Beckmann N. (2013). Targeting Iron Acquisition Blocks Infection with the Fungal Pathogens *Aspergillus fumigatus* and *Fusarium oxysporum*. PLoS Pathog..

[39] Lindahl P.A. (2019). A comprehensive mechanistic model of iron metabolism in: *Saccharomyces cerevisiae*. Metallomics.

[40] Almeida R.S., Wilson D., Hube B. (2009). *Candida albicans* iron acquisition within the host. FEMS Yeast Res..

[41] Gaensly F., Picheth G., Brand D., Bonfim T.M.B. (2014). The uptake of different iron salts by the yeast *Saccharomyces cerevisiae*. Braz. J. Microbiol..

[42] Walther G., Wagner L., Kurzai O. (2019). Updates on the taxonomy of mucorales with an emphasis on clinically important taxa. J. Fungi.

[43] Gerwien F., Skrahina V., Kasper L., Hube B., Brunke S. (2018). Metals in fungal virulence. FEMS Microbiol. Rev..

[44] Knight S.A.B., Lesuisse E., Stearman R., Klausner R.D., Dancis A. (2002). Reductive iron uptake by *Candida albicans*: Role of copper, iron and the TUP1 regulator. Microbiology.

[45] Knight S.A.B., Vilaire G., Lesuisse E., Dancis A. (2005). Iron Acquisition from Transferrin by *Candida albicans* Depends on the Reductive Pathway. Infect. Immun..

[46] Blatzer M., Binder U., Haas H. (2011). The metalloreductase FreB is involved in adaptation of *Aspergillus fumigatus* to iron starvation. Fungal Genet. Biol..

[47] Navarro-Mendoza M.I., Pérez-Arques C., Murcia L., Martínez-García P., Lax C., Sanchis M., Capilla J., Nicolás F.E., Garre V. (2018). Components of a new gene family of ferroxidases involved in virulence are functionally specialized in fungal dimorphism. Sci. Rep..

[48] Andrianaki A.M., Kyrmizi I., Thanopoulou K., Baldin C., Drakos E., Soliman S.S.M., Shetty A.C., McCracken C., Akoumianaki T., Stylianou K. (2018). Iron restriction inside macrophages regulates pulmonary host defense against *Rhizopus* species. Nat. Commun..

[49] Schwartze V.U., Winter S., Shelest E., Marcet-Houben M., Horn F., Wehner S., Linde J., Valiante V., Sammeth M., Riege K. (2014). Gene Expansion Shapes Genome Architecture in the Human Pathogen *Lichtheimia corymbifera*: An Evolutionary Genomics Analysis in the Ancient Terrestrial Mucorales (Mucoromycotina). PLoS Genet..

[50] Urbanowski J.L., Piper R.C. (1999). The iron transporter Fth1p forms a complex with the Fet5 iron oxidase and resides on the vacuolar membrane. J. Biol. Chem..

[51] Haas H., Petrik M., Decristoforo C. (2015). An Iron-Mimicking, Trojan Horse-Entering Fungi—Has the Time Come for Molecular Imaging of Fungal Infections?. PLoS Pathog..

[52] Saikia S., Oliveira D., Hu G., Kronstad J. (2014). Role of ferric reductases in iron acquisition and virulence in the fungal pathogen *Cryptococcus neoformans*. Infect. Immun..

[53] Georgatsou E., Mavrogiannis L.A., Fragiadakis G.S., Alexandraki D. (1997). The yeast Fre1p/Fre2p cupric reductases facilitate copper uptake and are regulated by the copper-modulated Mac1p activator. J. Biol. Chem..

[54] Georgatsou E., Alexandraki D. (1999). Regulated expression of the *Saccharomyces cerevisiae* Fre1p/Fre2p Fe/Cu reductase related genes. Yeast.

[55] Philpott C.C., Protchenko O. (2008). Response to iron deprivation in *Saccharomyces cerevisiae*. Eukaryot. Cell.

[56] Sun T.S., Ju X., Gao H.L., Wang T., Thiele D.J., Li J.Y., Wang Z.Y., Ding C. (2014). Reciprocal functions of *Cryptococcus neoformans* copper homeostasis machinery during pulmonary infection and meningoencephalitis. Nat. Commun..

[57] Waterman S.R., Hacham M., Hu G., Zhu X., Park Y.D., Shin S., Panepinto J., Valyi-Nagy T., Beam C., Husain S. (2007). Role of a CUF1/CTR4 copper regulatory axis in the virulence of *Cryptococcus neoformans*. J. Clin. Investig..

[58] Yamaguchi-Iwai Y., Serpe M., Haile D., Yang W., Kosman D.J., Klausner R.D., Dancis A. (1997). Homeostatic regulation of copper uptake in yeast via direct binding of MAC1 protein to upstream regulatory sequences of FRE1 and CTR1. J. Biol. Chem..

[59] Kwok E.Y., Severance S., Kosman D.J. (2006). Evidence for iron channeling in the Fet3p-Ftr1p high-affinity iron uptake complex in the yeast plasma membrane. Biochemistry.

[60] Bairwa G., Hee Jung W., Kronstad J.W. (2017). Iron acquisition in fungal pathogens of humans. Metallomics.

[61] Protchenko O., Ferea T., Rashford J., Tiedeman J., Brown P.O., Botstein D., Philpott C.C. (2001). Three Cell Wall Mannoproteins Facilitate the Uptake of Iron in *Saccharomyces cerevisiae*. J. Biol. Chem..

[62] Cheng X., Xu N., Yu Q., Ding X., Qian K., Zhao Q., Wang Y., Zhang B., Xing L., Li M. (2013). Novel insight into the expression and function of the multicopper oxidases in *Candida albicans*. Microbiology.

[63] Ramanan N., Wang Y. (2000). A high-affinity iron permease essential for *Candida albicans* virulence. Science.

[64] Pradhan A., Avelar G.M., Bain J.M., Childers D., Pelletier C., Larcombe D.E., Shekhova E., Netea M.G., Brown G.D., Erwig L. (2019). Non-canonical signalling mediates changes in fungal cell wall PAMPs that drive immune evasion. Nat. Commun..

[65] Yun C.W., Bauler M., Moore R.E., Klebba P.E., Philpott C.C. (2001). The Role of the FRE Family of Plasma Membrane Reductases in the Uptake of Siderophore-Iron in *Saccharomyces cerevisiae*. J. Biol. Chem..

[66] Fu Y., Lee H., Collins M., Tsai H.F., Spellberg B., Edwards J.E., Kwon-Chung K.J., Ibrahim A.S. (2004). Cloning and functional characterization of the *Rhizopus oryzae* high affinity iron permease (rFTR1) gene. FEMS Microbiol. Lett..

[67] Trieu T.A., Navarro-Mendoza M.I., Pérez-Arques C., Sanchis M., Capilla J., Navarro-Rodriguez P., Lopez-Fernandez L., Torres-Martínez S., Garre V., Ruiz-Vázquez R.M. (2017). RNAi-Based Functional Genomics Identifies New Virulence Determinants in Mucormycosis. PLoS Pathog..

[68] Singh A., Severance S., Kaur N., Wiltsie W., Kosman D.J. (2006). Assembly, activation, and trafficking of the Fet3p·Ftr1p high affinity iron permease complex in *Saccharomyces cerevisiae*. J. Biol. Chem..

[69] Severance S., Chakraborty S., Kosman D.J. (2004). The Ftr1p iron permease in the yeast plasma membrane: Orientation, topology and structure-function relationships. Biochem. J..

[70] Stearman R., Yuan D.S., Yamaguchi-Iwai Y., Klausner R.D., Dancis A. (1996). A Permease-Oxidase Complex Involved in High-Affinity Iron Uptake in Yeast. Science.

[71] Liu M., Lin L., Gebremariam T., Luo G., Skory C.D., French S.W., Chou T.-F., Edwards J.E., Ibrahim A.S. (2015). Fob1 and Fob2 Proteins Are Virulence Determinants of *Rhizopus oryzae* via Facilitating Iron Uptake from Ferrioxamine. PLOS Pathog..

[72] Ibrahim A.S., Gebremariam T., Lin L., Luo G., Husseiny M.I., Skory C.D., Fu Y., French S.W., Edwards J.E., Spellberg B. (2010). The high affinity iron permease is a key virulence factor required for *Rhizopus oryzae* pathogenesis. Mol. Microbiol..

[73] López-Fernández L., Sanchis M., Navarro-Rodríguez P., Nicolás F.E., Silva-Franco F., Guarro J., Garre V., Navarro-Mendoza M.I., Pérez-Arques C., Capilla J. (2018). Understanding *Mucor circinelloides* pathogenesis by comparative genomics and phenotypical studies. Virulence.

[74] Schrettl M., Bignell E., Kragl C., Joechl C., Rogers T., Arst H.N., Haynes K., Haas H. (2004). Siderophore Biosynthesis But Not Reductive Iron Assimilation Is Essential for *Aspergillus fumigatus* Virulence. J. Exp. Med..

[75] Schrettl M., Bignell E., Kragl C., Sabiha Y., Loss O., Eisendle M., Wallner A., Arst H.N., Haynes K., Haas H. (2007). Distinct roles for intra- and extracellular siderophores during *Aspergillus fumigatus* infection. PLoS Pathog..

[76] Gerwien F., Safyan A., Wisgott S., Brunke S., Kasper L., Hube B. (2017). The Fungal Pathogen *Candida glabrata* Does not Depend on Surface Ferric Reductases for Iron Acquisition. Front. Microbiol..

[77] Aisen P., Leibman A., Zweier J. (1978). Stoichiometric and site characteristics of the binding of iron to human transferrin. J. Biol. Chem..

[78] Hare S.A. (2017). Diverse structural approaches to haem appropriation by pathogenic bacteria. Biochim. Biophys. Acta-Proteins Proteom..

[79] Nairz M., Schroll A., Sonnweber T., Weiss G. (2010). The struggle for iron - a metal at the host-pathogen interface. Cell. Microbiol..

[80] Meynard D., Babitt J.L., Lin H.Y. (2013). Review Article The liver: Conductor of systemic iron balance. Blood.

[81] Weissman Z., Kornitzer D. (2004). A family of *Candida* cell surface haem-binding proteins involved in haemin and haemoglobin-iron utilization. Mol. Microbiol..

[82] Devaux F., Thiébaut A. (2019). The regulation of iron homeostasis in the fungal human pathogen *Candida glabrata*. Microbiology.

[83] Fourie R., Kuloyo O.O., Mochochoko B.M., Albertyn J., Pohl C.H. (2018). Iron at the centre of *Candida albicans* interactions. Front. Cell. Infect. Microbiol..

[84] Santos R., Buisson N., Knight S., Dancis A., Camadro J., Lesuisse E., Inge L. (2003). Haemin uptake and use as an iron source by *Candida albicans*: Role of CaHMX1 -encoded haem oxygenase. Microbiology.

[85] Weissman Z., Shemer R., Conibear E., Kornitzer D. (2008). An endocytic mechanism for haemoglobin-iron acquisition in *Candida albicans*. Mol. Microbiol..

[86] Ding C., Vidanes G.M., Maguire S.L., Guida A., Synnott J.M., Andes D.R., Butler G. (2011). Conserved and Divergent Roles of Bcr1 and CFEM Proteins in *Candida parapsilosis* and *Candida albicans*. PLoS ONE.

[87] Okamoto-Shibayama K., Kikuchi Y., Kokubu E., Sato Y., Ishihara K. (2014). Csa2, a member of the Rbt5 protein family, is involved in the utilization of iron from human hemoglobin during *Candida albicans* hyphal growth. FEMS Yeast Res..

[88] Kulkarni R., Kelkar H., Dean R. (2003). An eight-cysteine-containing CFEM domain unique toa group of fungal membrane proteins. Trends Biochem. Sci..

[89] Plaine A., Walker L., Da Costa G., Mora-Montes H.M., McKinnon A., Gow N.A.R., Gaillardin C., Munro C.A., Richard M.L. (2008). Functional analysis of Candida albicans GPI-anchored proteins: Roles in cell wall integrity and caspofungin sensitivity. Fungal Genet. Biol..

[90] Kuznets G., Vigonsky E., Weissman Z., Lalli D., Gildor T., Kauffman S.J., Turano P., Becker J., Lewinson O., Kornitzer D. (2014). A Relay Network of Extracellular Heme-Binding Proteins Drives *C. albicans* Iron Acquisition from Hemoglobin. PLoS Pathog..

[91] Pinsky M., Roy U., Moshe S., Weissman Z., Kornitzer D. (2020). Human Serum Albumin Facilitates Heme-Iron Utilization by Fungi. MBio.

[92] Caza M., Kronstad J.W. (2013). Shared and distinct mechanisms of iron acquisition by bacterial and fungal pathogens of humans. Front. Cell. Infect. Microbiol..

[93] Nevitt T., Thiele D.J. (2011). Host iron withholding demands siderophore utilization for *Candida glabrata* to survive macrophage killing. PLoS Pathog..

[94] Srivastava V.K., Suneetha K.J., Kaur R. (2014). A systematic analysis reveals an essential role for high-affinity iron uptake system, haemolysin and CFEM domain-containing protein in iron homoeostasis and virulence in *Candida glabrata*. Biochem. J..

[95] Karkowska-Kuleta J., Satala D., Bochenska O., Rapala-Kozik M., Kozik A. (2019). Moonlighting proteins are variably exposed at the cell surfaces of *Candida glabrata*, *Candida parapsilosis* and *Candida tropicalis* under certain growth conditions. BMC Microbiol..

[96] Hu G., Caza M., Cadieux B., Bakkeren E., Do E., Jung W.H., Kronstad J.W. (2015). The endosomal sorting complex required for transport machinery influences haem uptake and capsule elaboration in *Cryptococcus neoformans*. Mol. Microbiol..

[97] Kim J., Cho Y.-J., Do E., Choi J., Hu G., Cadieux B., Chun J., Lee Y., Kronstad J.W., Jung W.H. (2012). A defect in iron uptake enhances the susceptibility of *Cryptococcus neoformans* to azole antifungal drugs. Fungal Genet. Biol..

[98] Cadieux B., Lian T., Hu G., Wang J., Biondo C., Teti G., Liu V., Murphy M.E.P., Creagh A.L., Kronstad J.W. (2013). The mannoprotein cig1 supports iron acquisition from heme and virulence in the pathogenic fungus *Cryptococcus neoformans*. J. Infect. Dis..

[99] Hu G., Caza M., Cadieux B., Chan V., Liu V., Kronstad J. (2013). *Cryptococcus neoformans* requires the ESCRT protein Vps23 for iron acquisition from heme, for capsule formation, and for virulence. Infect. Immun..

[100] Bairwa G., Caza M., Horianopoulos L., Hu G., Kronstad J. (2019). Role of clathrin-mediated endocytosis in the use of heme and hemoglobin by the fungal pathogen *Cryptococcus neoformans*. Cell. Microbiol..

[101] Spellberg B., Walsh T.J., Kontoyiannis D.P., Edwards J., Ibrahim A.S. (2009). Recent Advances in the Management of Mucormycosis: From Bench to Bedside. Clin. Infect. Dis..

[102] Schwartze V.U., Hoffmann K., Nyilasi I., Papp T., Vágvölgyi C., de Hoog S., Voigt K., Jacobsen I.D. (2012). *Lichtheimia* species exhibit differences in virulence potential. PLoS ONE.

[103] Lesuisse E., Blaiseau P.L., Dancis A., Camadro J.M. (2001). Siderophore uptake and use by the yeast *Saccharomyces cerevisiae*. Microbiology.

[104] Wilson B.R., Bogdan A.R., Miyazawa M., Hashimoto K., Tsuji Y. (2016). Siderophores in Iron Metabolism: From Mechanism to Therapy Potential. Trends Mol. Med..

[105] Baldin C., Ibrahim A.S. (2017). Molecular mechanisms of mucormycosis—The bitter and the sweet. PLoS Pathog..

[106] Larcher G., Dias M., Razafimandimby B., Bomal D., Bouchara J.-P. (2013). Siderophore Production by Pathogenic Mucorales and Uptake of Deferoxamine B. Mycopathologia.

[107] Butler A., Theisen R.M. (2010). Iron(III)–siderophore coordination chemistry: Reactivity of marine siderophores. Coord. Chem. Rev..

[108] Kurth C., Kage H., Nett M. (2016). Siderophores as molecular tools in medical and environmental applications. Org. Biomol. Chem..

[109] Saha M., Sarkar S., Sarkar B., Sharma B.K., Bhattacharjee S., Tribedi P. (2016). Microbial siderophores and their potential applications: A review. Environ. Sci. Pollut. Res..

[110] Howard D.H. (1999). Acquisition, transport, and storage of iron by pathogenic fungi. Clin. Microbiol. Rev..

[111] Miethke M., Marahiel M.A. (2007). Siderophore-Based Iron Acquisition and Pathogen Control. Microbiol. Mol. Biol. Rev..

[112] Hider R.C., Kong X. (2010). Chemistry and biology of siderophores. Nat. Prod. Rep..

[113] Neilands J.B. (1995). Siderophores: Structure and function of microbial iron transport compounds. J. Biol. Chem..

[114] Haas H., Schoeser M., Lesuisse E., Ernst J.F., Parson W., Abt B. (2003). Characterization of the *Aspergillus nidulans* transporters for the siderophores enterobactin and triacetylfusarinine C. Biochem. J..

[115] De Locht M., Boelaert J.R., Schneider Y.-J. (1994). Iron uptake from ferrioxamine and from ferrirhizoferrin by germinating spores of *Rhizopus microsporus*. Biochem. Pharmacol..

[116] Coale T.H., Moosburner M., Horák A., Oborník M., Barbeau K.A., Allen A.E. (2019). Reduction-dependent siderophore assimilation in a model pennate diatom. Proc. Natl. Acad. Sci. USA.

[117] Haas H. (2003). Molecular genetics of fungal siderophore biosynthesis and uptake: The role of siderophores in iron uptake and storage. Appl. Microbiol. Biotechnol..

[118] Yun C., Tiedeman J.S., Moore R.E., Philpott C.C. (2000). Siderophore-iron uptake in *Saccharomyces cerevisiae*: Identification of ferrichrome and fusarinine transporters. J. Biol. Chem..

[119] Kim Y. (2002). Ferrichrome induces endosome to plasma membrane cycling of the ferrichrome transporter, Arn1p, in *Saccharomyces cerevisiae*. EMBO J..

[120] Froissard M., Belgareh-touzé N., Dias M., Buisson N., Camadro J.M., Haguenauer-tsapis R., Lesuisse E. (2007). Trafficking of siderophore transporters in *Saccharomyces cerevisiae* and intracellular fate of ferrioxamine B conjugates. Traffic.

[121] Moore R.E., Kim Y., Philpott C.C. (2003). The mechanism of ferrichrome transport through Arn1p and its metabolism in *Saccharomyces cerevisiae*. Proc. Natl. Acad. Sci. USA.

[122] Nies D.H., Janssen P.J., Van Houdt R., Moors H., Monsieurs P., Morin N., Michaux A., Benotmane M.A., Leys N., Vallaeys T. (2016). The biological chemistry of the transition metal “transportome” of *Cupriavidus metallidurans*. Metallomics.

[123] Wilson S., Bird A.J. (2016). Zinc sensing and regulation in yeast model systems. Arch. Biochem. Biophys..

[124] Howard D.H. (2004). Iron gathering by zoopathogenic fungi. FEMS Immunol. Med. Microbiol..

[125] Heymann P., Gerads M., Schaller M., Dromer F., Winkelmann G., Ernst J.F. (2002). The siderophore iron transporter of *Candida albicans* (Sit1p/Arn1p) mediates uptake of ferrichrome-type siderophores and is required for epithelial invasion. Infect. Immun..

[126] Heymann P., Ernst J.F., Winkelmann G. (2000). A gene of the major facilitator superfamily encodes a transporter for enterobactin (Enb1p) in *Saccharomyces cerevisiae*. BioMetals.

[127] Castresana J. (2000). Selection of conserved blocks from multiple alignments for their use in phylogenetic analysis. Mol. Biol. Evol..

[128] Guindon S., Gascuel O. (2003). A Simple, Fast, and Accurate Algorithm to Estimate Large Phylogenies by Maximum Likelihood. Syst. Biol..

[129] Edgar R.C. (2004). MUSCLE: Multiple sequence alignment with high accuracy and high throughput. Nucleic Acids Res..

[130] Anisimova M., Gascuel O. (2006). Approximate likelihood-ratio test for branches: A fast, accurate, and powerful alternative. Syst. Biol..

[131] Chevenet F., Brun C., Bañuls A.L., Jacq B., Christen R. (2006). TreeDyn: Towards dynamic graphics and annotations for analyses of trees. BMC Bioinform..

[132] Dereeper A., Guignon V., Blanc G., Audic S., Buffet S., Chevenet F., Dufayard J.-F., Guindon S., Lefort V., Lescot M. (2008). Phylogeny.fr: Robust phylogenetic analysis for the non-specialist. Nucleic Acids Res..

[133] Dereeper A., Audic S., Claverie J.-M., Blanc G. (2010). BLAST-EXPLORER helps you building datasets for phylogenetic analysis. BMC Evol. Biol..

[134] Jeong M., Kang C., Kim J., Heo D., Chang M., Baek I., Ro H., Choi I.-D., Kim T.-H., Yun C.-W. (2009). A novel function of Aft1 in regulating ferrioxamine B uptake: Aft1 modulates Arn3 ubiquitination in *Saccharomyces cerevisiae*. Biochem. J..

[135] Kang C.-M., Kang S., Park Y., Yun C. (2015). Physical interaction between Sit1 and Aft1 upregulates FOB uptake activity by inhibiting protein degradation of Sit1 in *Saccharomyces cerevisiae*. FEMS Yeast Res..

[136] Noble S.M. (2013). *Candida albicans* specializations for iron homeostasis: From commensalism to virulence. Curr. Opin. Microbiol..

[137] Seider K., Gerwien F., Kasper L., Allert S., Brunke S., Jablonowski N., Schwarzmüller T., Barz D., Rupp S., Kuchler K. (2014). Immune evasion, stress resistance, and efficient nutrient acquisition are crucial for intracellular survival of *Candida glabrata* within macrophages. Eukaryot. Cell.

[138] Jung W.H., Sham A., Lian T., Singh A., Kosman D.J., Kronstad J.W. (2008). Iron Source Preference and Regulation of Iron Uptake in *Cryptococcus neoformans*. PLoS Pathog..

[139] Tangen K.L., Jung W.H., Sham A.P., Lian T., Kronstad J.W. (2007). The iron- and cAMP-regulated gene SIT1 influences ferrioxamine B utilization, melanization and cell wall structure in *Cryptococcus neoformans*. Microbiology.

[140] Kennedy K.J., Daveson K., Slavin M.A., van Hal S.J., Sorrell T.C., Lee A., Marriott D.J., Chapman B., Halliday C.L., Hajkowicz K. (2016). Mucormycosis in Australia: Contemporary epidemiology and outcomes. Clin. Microbiol. Infect..

[141] Chow V., Khan S., Balogun A., Mitchell D., Mühlschlegel F.A. (2015). Invasive rhino-orbito-cerebral mucormycosis in a diabetic patient—The need for prompt treatment. Med. Mycol. Case Rep..

[142] Boelaert J.R., De Locht M., Van Cutsem J., Kerrels V., Cantinieaux B., Verdonck A., Van Landuyt H.W., Schneider Y.J. (1993). Mucormycosis during deferoxamine therapy is a siderophore-mediated infection: In vitro and in vivo animal studies. J. Clin. Investig..

[143] Boelaert J.R., Van Cutsem J., De Locht M., Schneider Y.J., Crichton R.R. (1994). Deferoxamine augments growth and pathogenicity of *Rhizopus*, while hydroxypyridinone chelators have no effect. Kidney Int..

[144] Van Cutsem J., Boelaert J.R. (1989). Effects of deferoxamine, feroxamine and iron on experimental mucormycosis (zygomycosis). Kidney Int..

[145] Alastruey-Izquierdo A., Hoffmann K., De Hoog G.S., Rodriguez-Tudela J.L., Voigt K., Bibashi E., Walther G. (2010). Species recognition and clinical relevance of the zygomycetous genus *Lichtheimia* (syn. *Absidia* pro parte, Mycocladus). J. Clin. Microbiol..

[146] Schrettl M., Ibrahim-Granet O., Droin S., Huerre M., Latgé J.P., Haas H. (2010). The crucial role of the *Aspergillus fumigatus* siderophore system in interaction with alveolar macrophages. Microbes Infect..

[147] Hissen A.H.T., Wan A.N.C., Warwas M.L., Pinto L.J., Moore M.M. (2005). The *Aspergillus fumigatus* Siderophore Biosynthetic Gene sidA, Encoding l-Ornithine N5-Oxygenase, Is Required for Virulence. Infect. Immun..

[148] Schrettl M., Kim H.S., Eisendle M., Kragl C., Nierman W.C., Heinekamp T., Werner E.R., Jacobsen I., Illmer P., Yi H. (2008). SreA-mediated iron regulation in *Aspergillus fumigatus*. Mol. Microbiol..

[149] Burt W.R. (1982). Identification of coprogen B and its breakdown products from *Histoplasma capsulatum*. Infect. Immun..

[150] Silva-Bailão M.G., Bailão E.F.L.C., Lechner B.E., Gauthier G.M., Lindner H., Bailão A.M., Haas H., Soares C.M.D.A. (2014). Hydroxamate production as a high affinity iron acquisition mechanism in *Paracoccidioides* spp.. PLoS ONE.

[151] Hwang L.H., Mayfield J.A., Rine J., Sil A. (2008). *Histoplasma* requires SID1, a member of an iron-regulated siderophore gene cluster, for host colonization. PLoS Pathog..

[152] Beckmann N., Schafferer L., Schrettl M., Binder U., Talasz H., Lindner H., Haas H. (2013). Characterization of the Link between Ornithine, Arginine, Polyamine and Siderophore Metabolism in *Aspergillus fumigatus*. PLoS ONE.

[153] Park Y.S., Kim J.Y., Yun C.W. (2016). Identification of ferrichrome- and ferrioxamine B-mediated iron uptake by *Aspergillus fumigatus*. Biochem. J..

[154] Hwang L.H., Seth E., Gilmore S.A., Sil A. (2012). SRE1 Regulates Iron-Dependent and -Independent Pathways in the Fungal Pathogen *Histoplasma capsulatum*. Eukaryot. Cell.

[155] Lima S.L., Colombo A.L., de Almeida Junior J.N. (2019). Fungal Cell Wall: Emerging Antifungals and Drug Resistance. Front. Microbiol..

[156] Drummond R.A., Gaffen S.L., Hise A.G., Brown G.D. (2015). Innate defense against fungal pathogens. Cold Spring Harb. Perspect. Med..

[157] Gow N.A.R., Latge J., Munro C.A. (2017). The Fungal Cell Wall: Structure, Biosynthesis, and Function. Microbiol. Spectr..

[158] Arana D.M., Prieto D., Román E., Nombela C., Alonso-Monge R., Pla J. (2009). The role of the cell wall in fungal pathogenesis. Microb. Biotechnol..

[159] Bayry J., Beaussart A., Dufrêne Y.F., Sharma M., Bansal K., Kniemeyer O., Aimanianda V., Brakhage A.A., Kaveri S.V., Kwon-Chung K.J. (2014). Surface structure characterization of *Aspergillus fumigatus* conidia mutated in the melanin synthesis pathway and their human cellular immune response. Infect. Immun..

[160] Latgé J.P., Beauvais A. (2014). Functional duality of the cell wall. Curr. Opin. Microbiol..

[161] de Carrion S.J., Leal S.M., Ghannoum M.A., Aimanianda V., Latgé J.-P., Pearlman E. (2013). The RodA Hydrophobin on *Aspergillus fumigatus* Spores Masks Dectin-1– and Dectin-2–Dependent Responses and Enhances Fungal Survival In Vivo. J. Immunol..

[162] Casadevall A., Rosas A.L., Nosanchuk J.D. (2000). Melanin and virulence in *Cryptococcus neoformans*. Curr. Opin. Microbiol..

[163] Wheeler R.T., Fink G.R. (2006). A drug-sensitive genetic network masks fungi from the immune system. PLoS Pathog..

[164] Wheeler R.T., Kombe D., Agarwala S.D., Fink G.R. (2008). Dynamic, morphotype-specific *Candida albicans* β-glucan exposure during infection and drug treatment. PLoS Pathog..

[165] Bozza S., Clavaud C., Giovannini G., Fontaine T., Beauvais A., Sarfati J., D’Angelo C., Perruccio K., Bonifazi P., Zagarella S. (2009). Immune Sensing of *Aspergillus fumigatus* Proteins, Glycolipids, and Polysaccharides and the Impact on Th Immunity and Vaccination. J. Immunol..

[166] Ram A.F.J., Kapteyn J.C., Montijn R.C., Caro L.H.P., Douwes J.E., Baginsky W., Mazur P., van den Ende H., Klis F.M. (1998). Loss of the Plasma Membrane-Bound Protein Gas1p in *Saccharomyces cerevisiae* Results in the Release of β1,3-Glucan into the Medium and Induces a Compensation Mechanism To Ensure Cell Wall Integrity. J. Bacteriol..

[167] Cortés J.C.G., Curto M.Á., Carvalho V.S.D., Pérez P., Ribas J.C. (2019). The fungal cell wall as a target for the development of new antifungal therapies. Biotechnol. Adv..

[168] Garcia-Rubio R., de Oliveira H.C., Rivera J., Trevijano-Contador N. (2020). The Fungal Cell Wall: *Candida*, *Cryptococcus*, and *Aspergillus* Species. Front. Microbiol..

[169] Casadevall A., Nosanchuk J.D., Williamson P., Rodrigues M.L. (2009). Vesicular transport across the fungal cell wall. Trends Microbiol..

[170] Erwig L.P., Gow N.A.R. (2016). Interactions of fungal pathogens with phagocytes. Nat. Rev. Microbiol..

[171] Lecointe K., Cornu M., Leroy J., Coulon P., Sendid B. (2019). Polysaccharides cell wall architecture of mucorales. Front. Microbiol..

[172] Synytsya A., Novak M. (2014). Structural analysis of glucans. Ann. Transl. Med..

[173] Masuoka J. (2004). Surface Glycans of *Candida albicans* and Other Pathogenic Fungi: Physiological Roles, Clinical Uses, and Experimental Challenges. Clin. Microbiol. Rev..

[174] Beauvais A., Bozza S., Kniemeyer O., Formosa C., Balloy V., Henry C., Roberson R.W., Dague E., Chignard M., Brakhage A.A. (2013). Deletion of the α-(1,3)-Glucan Synthase Genes Induces a Restructuring of the Conidial Cell Wall Responsible for the Avirulence of *Aspergillus fumigatus*. PLoS Pathog..

[175] Mazur P., Baginsky W. (1996). In Vitro Activity of 1,3-β-D-Glucan Synthase Requires the GTP-binding Protein Rho1. Biochemistry.

[176] Aguilar-Zapata D., Petraitiene R., Petraitis V. (2015). Echinocandins: The Expanding Antifungal Armamentarium. Clin. Infect. Dis..

[177] Yoshimi A., Miyazawa K., Abe K. (2017). Function and biosynthesis of cell wall α-1,3-glucan in fungi. J. Fungi.

[178] Perlin D.S. (2007). Resistance to echinocandin-class antifungal drugs. Drug Resist. Updates.

[179] Lesage G., Bussey H. (2006). Cell Wall Assembly in *Saccharomyces cerevisiae*. Microbiol. Mol. Biol. Rev..

[180] Latgé J.P. (2007). The cell wall: A carbohydrate armour for the fungal cell. Mol. Microbiol..

[181] Chaudhary P.M., Tupe S.G., Deshpande M.V. (2013). Chitin Synthase Inhibitors as Antifungal Agents. Mini-Rev. Med. Chem..

[182] Ruiz-Herrera J., San-Blas G. (2003). Chitin synthesis as a target for antifungal drugs. Curr. Drug Targets-Infect. Disord..

[183] Sorrell T.C., Chen S.C.A. (2009). Fungal-derived immune modulating molecules. Adv. Exp. Med. Biol..

[184] Vecchiarelli A. (2000). Immunoregulation by capsular components of *Cryptococcus neoformans*. Med. Mycol..

[185] Engel J., Schmalhorst P.S., Routier F.H. (2012). Biosynthesis of the fungal cell wall polysaccharide galactomannan requires intraluminal GDP-mannose. J. Biol. Chem..

[186] Bowman S.M., Free S.J. (2006). The structure and synthesis of the fungal cell wall. BioEssays.

[187] Liu L., Tewari R.P., Williamson P.R. (1999). Laccase protects *Cryptococcus neoformans* from antifungal activity of alveolar macrophages. Infect. Immun..

[188] Nosanchuk J.D., Stark R.E., Casadevall A. (2015). Fungal melanin: What do we know about structure?. Front. Microbiol..

[189] Doering T.L. (2009). How Sweet it is! Cell Wall Biogenesis and Polysaccharide Capsule Formation in *Cryptococcus neoformans*. Annu. Rev. Microbiol..

[190] Casadevall A. (2007). Determinants of virulence in the pathogenic fungi. Fungal Biol. Rev..

[191] Eisenman H.C., Casadevall A. (2012). Synthesis and assembly of fungal melanin. Appl. Microbiol. Biotechnol..

[192] Wang Y., Aisen P., Casadevall A. (1995). *Cryptococcus neoformans* melanin and virulence: Mechanism of action. Infect. Immun..

[193] Zaragoza O. (2019). Basic principles of the virulence of *Cryptococcus*. Virulence.

[194] Barnes R.A. (2008). Early diagnosis of fungal infection in immunocompromised patients. J. Antimicrob. Chemother..

[195] De Pauw B., Walsh T.J., Donnelly J.P., Stevens D.A., Edwards J.E., Calandra T., Pappas P.G., Maertens J., Lortholary O., Kauffman C.A. (2008). Revised Definitions of Invasive Fungal Disease from the European Organization for Research and Treatment of Cancer/Invasive. Clin. Infect. Dis.

[196] Husain S., Sole A., Alexander B.D., Aslam S., Avery R., Benden C., Billaud E.M., Chambers D., Danziger-Isakov L., Fedson S. (2016). The 2015 International Society for Heart and Lung Transplantation Guidelines for the management of fungal infections in mechanical circulatory support and cardiothoracic organ transplant recipients: Executive summary. J. Hear. Lung Transplant..

[197] Formanek P.E., Dilling D.F. (2019). Advances in the Diagnosis and Management of Invasive Fungal Disease. Chest.

[198] Hof H. (2010). IFI = invasive fungal infections. What is that? A misnomer, because a non-invasive fungal infection does not exist!. Int. J. Infect. Dis..

[199] Carlesse F., Daudt L.E., Seber A., Dutra Á.P., Melo A.S.A., Simões B.P., Macedo C.R.D., Bonfim C., Benites E., Gregianin L. (2019). A consensus document for the clinical management of invasive fungal diseases in pediatric patients with hematologic cancer and/or undergoing hematopoietic stem cell transplantation in Brazilian medical centers. Braz. J. Infect. Dis..

[200] Sipsas N.V., Pagoni M.N., Kofteridis D.P., Meletiadis J., Vrioni G., Papaioannou M., Antoniadou A., Petrikkos G., Samonis G. (2018). Management of invasive fungal infections in adult patients with hematological malignancies in Greece during the financial crisis: Challenges and recommendations. J. Fungi.

[201] Arvanitis M., Anagnostou T., Fuchs B.B., Caliendo A.M., Mylonakis E. (2014). Molecular and nonmolecular diagnostic methods for invasive fungal infections. Clin. Microbiol. Rev..

[202] Sipsas N.V., Gamaletsou M.N., Anastasopoulou A., Kontoyiannis D.P. (2018). Therapy of mucormycosis. J. Fungi.

[203] Tarrand J.J., Lichterfeld M., Warraich I., Luna M., Han X.Y., May G.S., Kontoyiannis D.P. (2003). Diagnosis of invasive septate mold infections: A correlation of microbiological culture and histologic or cytologic examination. Am. J. Clin. Pathol..

[204] Cornely O.A., Arikan-Akdagli S., Dannaoui E., Groll A.H., Lagrou K., Chakrabarti A., Lanternier F., Pagano L., Skiada A., Akova M. (2014). ESCMID and ECMM joint clinical guidelines for the diagnosis and management of mucormycosis 2013. Clin. Microbiol. Infect..

[205] Colombo A.L., de Almeida Júnior J.N., Slavin M.A., Chen S.C.A., Sorrell T.C. (2017). Candida and invasive mould diseases in non-neutropenic critically ill patients and patients with haematological cancer. Lancet Infect. Dis..

[206] Bassetti M., Garnacho-Montero J., Calandra T., Kullberg B., Dimopoulos G., Azoulay E., Chakrabarti A., Kett D., Leon C., Ostrosky-Zeichner L. (2017). Intensive care medicine research agenda on invasive fungal infection in critically ill patients. Intensiv. Care Med..

[207] Sanguinetti M., Posteraro B., Beigelman-Aubry C., Lamoth F., Dunet V., Slavin M., Richardson M.D. (2019). Diagnosis and treatment of invasive fungal infections: Looking ahead. J. Antimicrob. Chemother..

[208] Lamoth F., Calandra T. (2017). Early diagnosis of invasive mould infections and disease. J. Antimicrob. Chemother..

[209] Galgóczy L. (2005). Molecular characterization of opportunistic pathogenic zygomycetes. Acta Biol. Szeged..

[210] Skiada A., Lass-Floerl C., Klimko N., Ibrahim A., Roilides E., Petrikkos G. (2018). Challenges in the diagnosis and treatment of mucormycosis. Med. Mycol..

[211] Oz Y., Kiraz N. (2011). Diagnostic methods for fungal infections in pediatric patients: Microbiological, serological and molecular methods. Expert Rev. Anti-Infect. Ther..

[212] Kontoyiannis D.P., Sumoza D., Tarrand J., Bodey G.P., Storey R., Raad I.I. (2000). Significance of aspergillemia in patients with cancer: A 10-year study. Clin. Infect. Dis..

[213] Marty F.M., Lowry C.M., Lempitski S.J., Kubiak D.W., Finkelman M.A., Baden L.R. (2006). Reactivity of (1→3)-β-D-glucan assay with commonly used intravenous antimicrobials. Antimicrob. Agents Chemother..

[214] Bajpai V.K., Khan I., Shukla S., Kumar P., Rather I.A., Park Y.H., Huh Y.S., Han Y.K. (2019). Invasive Fungal Infections and Their Epidemiology: Measures in the Clinical Scenario. Biotechnol. Bioprocess Eng..

[215] Schwartz S., Kontoyiannis D.P., Harrison T., Ruhnke M. (2018). Advances in the diagnosis and treatment of fungal infections of the CNS. Lancet Neurol..

[216] Zheng F., Zha H., Yang D., Deng J., Zhang Z. (2017). Diagnostic Values and Limitations of (1,3)-β-d-Glucans and Galactomannan Assays for Invasive Fungal Infection in Patients Admitted to Pediatric Intensive Care Unit. Mycopathologia.

[217] Bernal-Martínez L., Buitrago M.J., Castelli M.V., Rodriguez-Tudela J.L., Cuenca-Estrella M. (2013). Development of a single tube multiplex real-time PCR to detect the most clinically relevant Mucormycetes species. Clin. Microbiol. Infect..

[218] Schwarz P., Cornely O.A., Dannaoui E. (2019). Antifungal combinations in Mucorales: A microbiological perspective. Mycoses.

[219] White P.L., Alanio A., Cruciani M., Gorton R., Millon L., Rickerts V., Barnes R.A., Donnelly J.P., Loeffler J. (2020). Nucleic Acid Tools for Invasive Fungal Disease Diagnosis. Curr. Fungal Infect. Rep..

[220] Chong G.L.M., Van De Sande W.W.J., Dingemans G.J.H., Gaajetaan G.R., Vonk A.G., Hayette M.P., Van Tegelen D.W.E., Simons G.F.M., Rijnders B.J.A. (2015). Validation of a new *Aspergillus* real-time PCR assay for direct detection of *Aspergillus* and azole resistance of *Aspergillus fumigatus* on bronchoalveolar lavage fluid. J. Clin. Microbiol..

[221] Ellis M. (2002). Invasive fungal infections: Evolving challenges for diagnosis and therapeutics. Mol. Immunol..

[222] Tissot F., Agrawal S., Pagano L., Petrikkos G., Groll A.H., Skiada A., Lass-Flörl C., Calandra T., Viscoli C., Herbrecht R. (2017). ECIL-6 guidelines for the treatment of invasive candidiasis, aspergillosis and mucormycosis in leukemia and hematopoietic stem cell transplant patients. Haematologica.

[223] Petrikkos G., Skiada A., Lortholary O., Roilides E., Walsh T.J., Kontoyiannis D.P. (2012). Epidemiology and clinical manifestations of mucormycosis. Clin. Infect. Dis..

[224] Alastruey-Izquierdo A., Cuesta I., Walther G., Cuenca-Estrella M., Rodriguez-Tudela J.L. (2010). Antifungal susceptibility profile of human-pathogenic species of *Lichtheimia*. Antimicrob. Agents Chemother..

[225] Marquez L., Quave C.L. (2020). Prevalence and Therapeutic Challenges of Fungal Drug Resistance: Role for Plants in Drug Discovery. Antibiotics.

[226] Perfect J.R., Dismukes W.E., Dromer F., Goldman D.L., Graybill J.R., Hamill R.J., Harrison T.S., Larsen R.A., Lortholary O., Nguyen M.-H. (2010). Clinical Practice Guidelines for the Management of Cryptococcal Disease: 2010 Update by the Infectious Diseases Society of America. Clin. Infect. Dis..

[227] Mourad A., Perfect J. (2018). Present and Future Therapy of *Cryptococcus* Infections. J. Fungi.

[228] Marty F.M., Ostrosky-Zeichner L., Cornely O.A., Mullane K.M., Perfect J.R., Thompson G.R., Alangaden G.J., Brown J.M., Fredricks D.N., Heinz W.J. (2016). Isavuconazole treatment for mucormycosis: A single-arm open-label trial and case-control analysis. Lancet Infect. Dis..

[229] Walsh T.J., Lutsar I., Driscoll T., Dupont B., Roden M., Ghahramani P., Hodges M., Groll A.H., Perfect J.R. (2002). Voriconazole in the treatment of *Aspergillosis*, *Scedosporiosis* and other invasive fungal infections in children. Pediatr. Infect. Dis. J..

[230] Shoham S., Magill S.S., Merz W.G., Gonzalez C., Seibel N., Buchanan W.L., Knudsen T.A., Sarkisova T.A., Walsh T.J. (2010). Primary treatment of zygomycosis with liposomal amphotericin B: Analysis of 28 cases. Med. Mycol..

[231] Lax C., Pérez-Arques C., Navarro-Mendoza M.I., Cánovas-Márquez J.T., Tahiri G., Pérez-Ruiz J.A., Osorio-Concepción M., Murcia-Flores L., Navarro E., Garre V. (2020). Genes, Pathways, and Mechanisms Involved in the Virulence of Mucorales. Genes (Basel).

[232] Perkhofer S., Lechner V., Lass-Flörl C. (2009). In Vitro Activity of Isavuconazole against *Aspergillus* Species and Zygomycetes According to the Methodology of the European Committee on Antimicrobial Susceptibility Testing. Antimicrob. Agents Chemother..

[233] Pagano L., Caira M., Valentini C.G., Posteraro B., Fianchi L. (2010). Current therapeutic approaches to fungal infections in immunocompromised hematological patients. Blood Rev..

[234] Drew R. (2006). Potential role of aerosolized amphotericin B formulations in the prevention and adjunctive treatment of invasive fungal infections. Int. J. Antimicrob. Agents.

[235] Butts A., Palmer G.E., David Rogers P. (2016). Antifungal adjuvants: Preserving and extending the antifungal arsenal. Virulence.

[236] Holmes A.R., Cardno T.S., Strouse J.J., Ivnitski-Steele I., Keniya M.V., Lackovic K., Monk B.C., Sklar L.A., Cannon R.D. (2016). Targeting efflux pumps to overcome antifungal drug resistance. Futur. Med. Chem..

[237] Zarember K.A., Cruz A.R., Huang C.-Y., Gallin J.I. (2009). Antifungal Activities of Natural and Synthetic Iron Chelators Alone and in Combination with Azole and Polyene Antibiotics against *Aspergillus fumigatus*. Antimicrob. Agents Chemother..

[238] Walker L.A., Gow N.A.R., Munro C.A. (2013). Elevated chitin content reduces the susceptibility of *Candida* species to caspofungin. Antimicrob. Agents Chemother..

[239] Mansfield B.E., Oltean H.N., Oliver B.G., Hoot S.J., Leyde S.E., Hedstrom L., White T.C. (2010). Azole drugs are imported by facilitated diffusion in *Candida albicans* and other pathogenic fungi. PLoS Pathog..

[240] Prasad T., Chandra A., Mukhopadhyay C.K., Prasad R. (2006). Unexpected Link between Iron and Drug Resistance of *Candida* spp.: Iron Depletion Enhances Membrane Fluidity and Drug Diffusion, Leading to Drug-Susceptible Cells. Antimicrob. Agents Chemother..

[241] Ibrahim A.S., Gebermariam T., Fu Y., Lin L., Husseiny M.I., French S.W., Schwartz J., Skory C.D., Edwards J.E., Spellberg B.J. (2007). The iron chelator deferasirox protects mice from mucormycosis through iron starvation. J. Clin. Investig..

[242] Ibrahim A.S., Gebremariam T., Luo G., Fu Y., French S.W., Edwards J.E., Spellberg B. (2011). Combination Therapy of Murine Mucormycosis or *Aspergillosis* with Iron Chelation, Polyenes, and Echinocandins. Antimicrob. Agents Chemother..

[243] Spellberg B., Ibrahim A.S., Chin-Hong P.V., Kontoyiannis D.P., Morris M.I., Perfect J.R., Fredricks D., Brass E.P. (2012). The Deferasirox–AmBisome Therapy for Mucormycosis (DEFEAT Mucor) study: A randomized, double-blinded, placebo-controlled trial. J. Antimicrob. Chemother..

[244] Donnelly J.P., Lahav M. (2012). Deferasirox as adjunctive therapy for mucormycosis. J. Antimicrob. Chemother..

[245] Ibrahim A.S., Gebremariam T., French S.W., Edwards J.E., Spellberg B. (2009). The iron chelator deferasirox enhances liposomal amphotericin B efficacy in treating murine invasive pulmonary aspergillosis. J. Antimicrob. Chemother..

[246] Hassan M.I.A., Voigt K. (2019). Pathogenicity patterns of mucormycosis: Epidemiology, interaction with immune cells and virulence factors. Med. Mycol..

[247] Liu M., Spellberg B., Phan Q.T., Fu Y.Y., Fu Y.Y., Lee A.S., Edwards J.E., Filler S.G., Ibrahim A.S. (2010). The endothelial cell receptor GRP78 is required for mucormycosis pathogenesis in diabetic mice. J. Clin. Investig..

[248] Walker L.A., Gow N.A.R., Munro C.A. (2010). Fungal echinocandin resistance. Fungal Genet. Biol..

[249] Denning D.W. (2003). Echinocandin antifungal drugs. Lancet.

[250] Sucher A.J., Chahine E.B., Balcer H.E. (2009). Echinocandins: The newest class of antifungals. Ann. Pharmacother..

[251] Perlin D.S. (2015). Echinocandin Resistance in *Candida*. Clin. Infect. Dis..

[252] Onishi J., Meinz M., Thompson J., Curotto J., Dreikorn S., Rosenbach M., Douglas C., Abruzzo G., Flattery A., Kong L. (2000). Discovery of Novel Antifungal (1,3)-β-d-Glucan Synthase Inhibitors. Antimicrob. Agents Chemother..

[253] Hori Y., Shibuya K. (2018). Role of *FKS* Gene in the Susceptibility of Pathogenic Fungi to Echinocandins. Med. Mycol. J..

[254] Bowman J.C., Hicks P.S., Kurtz M.B., Rosen H., Schmatz D.M., Liberator P.A., Douglas C.M. (2002). The antifungal echinocandin caspofungin acetate kills growing cells of *Aspergillus fumigatus* in vitro. Antimicrob. Agents Chemother..

[255] Bachmann S.P., VandeWalle K., Ramage G., Patterson T.F., Wickes B.L., Graybill J.R., López-Ribot J.L. (2002). In vitro activity of caspofungin against *Candida albicans* biofilms. Antimicrob. Agents Chemother..

[256] Kuhn D.M., George T., Chandra J., Mukherjee P.K., Ghannoum M.A. (2002). Antifungal Susceptibility of *Candida* Biofilms: Unique Efficacy of Amphotericin B Lipid Formulations and Echinocandins. Antimicrob. Agents Chemother..

[257] Wagner C., Graninger W., Presterl E., Joukhadar C. (2006). The echinocandins: Comparison of their pharmacokinetics, pharmacodynamics and clinical applications. Pharmacology.

[258] Clancy C.J., Samanta P., Cheng S., Squires K., Nguyen M.-H. (2019). 1726. *Candida albicans* Virulence Genes Induced During Intra-abdominal Candidiasis (IAC) in the Absence of Antifungal Exposure Mediate Echinocandin Resistance. Open Forum Infect. Dis..

[259] Lee K.K., MacCallum D.M., Jacobsen M.D., Walker L.A., Odds F.C., Gow N.A.R., Munro C.A. (2012). Elevated cell wall chitin in *Candida albicans* confers echinocandin resistance in vivo. Antimicrob. Agents Chemother..

[260] Scorneaux B., Angulo D., Borroto-Esoda K., Ghannoum M., Peel M., Wring S. (2017). SCY-078 Is Fungicidal against *Candida* Species in Time-Kill Studies. Antimicrob. Agents Chemother..

[261] Larkin E.L., Long L., Isham N., Borroto-Esoda K., Barat S., Angulo D., Wring S., Ghannoum M. (2019). A novel 1,3-beta-D-glucan inhibitor, IbrexafungeRP (formerly SCY-078), shows potent activity in the lower pH environment of vulvovaginitis. Antimicrob. Agents Chemother..

[262] Seo K., Akiyoshi H., Ohnishi Y. (1999). Alteration of cell wall composition leads to amphotericin B resistance in *Aspergillus flavus*. Microbiol. Immunol..

[263] Mesa-Arango A.C., Rueda C., Román E., Quintin J., Terrón M.C., Luque D., Netea M.G., Pla J., Zaragoza O. (2016). Cell Wall Changes in Amphotericin B-Resistant Strains from *Candida tropicalis* and Relationship with the Immune Responses Elicited by the Host. Antimicrob. Agents Chemother..

[264] Kontoyiannis D.P., Lewis R.E. (2002). Antifungal drug resistance of pathogenic fungi. Lancet.

[265] Sheehan D.J., Hitchcock C.A., Sibley C.M. (1999). Current and emerging azole antifungal agents. Clin. Microbiol. Rev..

[266] Mast N., Zheng W., Stout C.D., Pikuleva I.A. (2013). Antifungal azoles: Structural insights into undesired tight binding to cholesterol-metabolizing cyp46a1s. Mol. Pharmacol..

[267] Perea S., Patterson T.F. (2002). Antifungal Resistance in Pathogenic Fungi. Clin. Infect. Dis..

[268] Berger S., Chazli Y.E., Babu A.F., Coste A.T. (2017). Azole resistance in *Aspergillus fumigatus*: A consequence of antifungal use in agriculture?. Front. Microbiol..

[269] Warrilow A.G., Nishimoto A.T., Parker J.E., Price C.L., Flowers S.A., Kelly D.E., David Rogers P., Kelly S.L. (2019). The evolution of Azole resistance in *Candida albicans* Sterol 14-demethylase (CYP51) through incremental amino acid substitutions. Antimicrob. Agents Chemother..

[270] Caramalho R., Tyndall J.D.A., Monk B.C., Larentis T., Lass-Flörl C., Lackner M. (2017). Intrinsic short-Tailed azole resistance in mucormycetes is due to an evolutionary conserved aminoacid substitution of the lanosterol 14α-demethylase. Sci. Rep..

[271] Vale-Silva L.A., Coste A.T., Ischer F., Parker J.E., Kelly S.L., Pinto E., Sanglard D. (2012). Azole Resistance by Loss of Function of the Sterol Δ 5,6 -Desaturase Gene (ERG3) in *Candida albicans* Does Not Necessarily Decrease Virulence. Antimicrob. Agents Chemother..

[272] Florio A., Ferrari S., De Carolis E., Torelli R., Fadda G., Sanguinetti M., Sanglard D., Posteraro B. (2011). Genome-wide expression profiling of the response to short-term exposure to fluconazole in *Cryptococcus neoformans* serotype A. BMC Microbiol..

[273] Martel C.M., Parker J.E., Warrilow A.G.S., Rolley N.J., Kelly S.L., Kelly D.E. (2010). Complementation of a *Saccharomyces cerevisiae* ERG11/CYP51 (sterol 14α-demethylase) doxycycline-regulated mutant and screening of the azole sensitivity of *Aspergillus fumigatus* isoenzymes CYP51A and CYP51B. Antimicrob. Agents Chemother..

[274] Lv Q.Z., Yan L., Jiang Y.Y. (2016). The synthesis, regulation, and functions of sterols in *Candida albicans*: Well-known but still lots to learn. Virulence.

[275] Bhattacharya S., Esquivel B.D., White T.C. (2018). Overexpression or Deletion of Ergosterol Biosynthesis Genes Alters Doubling Time, Response to Stress Agents, and Drug Susceptibility in *Saccharomyces cerevisiae*. MBio.

[276] Hameed S., Dhamgaye S., Singh A., Goswami S.K., Prasad R. (2011). Calcineurin Signaling and Membrane Lipid Homeostasis Regulates Iron Mediated MultiDrug Resistance Mechanisms in *Candida albicans*. PLoS ONE.

[277] Heilmann C.J., Schneider S., Barker K.S., Rogers P.D., Morschhäuser J. (2010). An A643T Mutation in the Transcription Factor Upc2p Causes Constitutive ERG11 Upregulation and Increased Fluconazole Resistance in *Candida albicans*. Antimicrob. Agents Chemother..

[278] Riemsma R., Hagen S., Kirschner-Hermanns R., Norton C., Wijk H., Andersson K.E., Chapple C., Spinks J., Wagg A., Hutt E. (2017). Can incontinence be cured? A systematic review of cure rates. BMC Med..

[279] Misslinger M., Gsaller F., Hortschansky P., Müller C., Bracher F., Bromley M.J., Haas H. (2017). The cytochrome: B 5 CybE is regulated by iron availability and is crucial for azole resistance in *A. fumigatus*. Metallomics.

[280] Sionov E., Chang Y.C., Garraffo H.M., Dolan M.A., Ghannoum M.A., Kwon-Chung K.J. (2012). Identification of a *Cryptococcus neoformans* Cytochrome P450 Lanosterol 14α-Demethylase (Erg11) Residue Critical for Differential Susceptibility between Fluconazole/Voriconazole and Itraconazole/Posaconazole. Antimicrob. Agents Chemother..

[281] Vitale R.G., De Hoog G.S., Schwarz P., Dannaoui E., Deng S., Machouart M., Voigt K., Van De Sande W.W.J.J., Dolatabadi S., Meis J.F. (2012). Antifungal susceptibility and phylogeny of opportunistic members of the order Mucorales. J. Clin. Microbiol..

[282] Alastruey-Izquierdo A., Castelli M.V., Cuesta I., Zaragoza O., Monzón A., Mellado E., Rodríguez-Tudela J.L. (2009). In vitro activity of antifungals against zygomycetes. Clin. Microbiol. Infect..

[283] Ritz N., Ammann R.A., Aebischer C.C., Gugger M., Jaton K., Schmid R.A., Aebi C. (2005). Failure of voriconazole to cure disseminated zygomycosis in an immunocompromised child. Eur. J. Pediatr..

[284] Almyroudis N.G., Sutton D.A., Fothergill A.W., Rinaldi M.G., Kusne S. (2007). In vitro susceptibilities of 217 clinical isolates of zygomycetes to conventional and new antifungal agents. Antimicrob. Agents Chemother..

